# A Novel Small-Molecule Inhibitor of Endosomal TLRs Reduces Inflammation and Alleviates Autoimmune Disease Symptoms in Murine Models

**DOI:** 10.3390/cells9071648

**Published:** 2020-07-09

**Authors:** Mahesh Chandra Patra, Asma Achek, Gi-Young Kim, Suresh Panneerselvam, Hyeon-Jun Shin, Wook-Yong Baek, Wang Hee Lee, June Sung, Uisuk Jeong, Eun-Young Cho, Wook Kim, Eunha Kim, Chang-Hee Suh, Sangdun Choi

**Affiliations:** 1 Department of Molecular Science and Technology, Ajou University, Suwon 16499, Korea; ml2mahesh@gmail.com (M.C.P.); asma.achek@gmail.com (A.A.); kimki276@naver.com (G.-Y.K.); sureshcbt@gmail.com (S.P.); shinex1212@nate.com (H.-J.S.); haneul246@ajou.ac.kr (W.H.L.); tjdwkf12@ajou.ac.kr (J.S.); dmltjr11@ajou.ac.kr (U.J.); whdmsdud8@naver.com (E.-Y.C.); wookkim21@ajou.ac.kr (W.K.); ehkim01@ajou.ac.kr (E.K.); 2 Department of Rheumatology, Ajou University School of Medicine, Suwon 16499, Korea; arikato83@naver.com (W.-Y.B.); chsuh@ajou.ac.kr (C.-H.S.)

**Keywords:** Toll-like receptor, endosomal TLR, antagonist, QSAR, TAC5, autoimmune diseases

## Abstract

Toll-like receptors (TLRs) play a fundamental role in the inflammatory response against invading pathogens. However, the dysregulation of TLR-signaling pathways is implicated in several autoimmune/inflammatory diseases. Here, we show that a novel small molecule TLR-inhibitor (TAC5) and its derivatives TAC5-a, TAC5-c, TAC5-d, and TAC5-e predominantly antagonized poly(I:C) (TLR3)-, imiquimod (TLR7)-, TL8-506 (TLR8)-, and CpG-oligodeoxynucleotide (TLR9)-induced signaling pathways. TAC5 and TAC5-a significantly hindered the activation of nuclear factor kappa-light-chain-enhancer of activated B cells (NF-κB), reduced the phosphorylation of mitogen-activated protein kinases, and inhibited the secretion of tumor necrosis factor-α (TNF-α) and interleukin-6. Besides, TAC5-a prevented the progression of psoriasis and systemic lupus erythematosus (SLE) in mice. Interestingly, TAC5 and TAC5-a did not affect Pam_3_CSK_4_ (TLR1/2)-, FSL-1 (TLR2/6)-, or lipopolysaccharide (TLR4)-induced TNF-α secretion, indicating their specificity towards endosomal TLRs (TLR3/7/8/9). Collectively, our data suggest that the TAC5 series of compounds are potential candidates for treating autoimmune diseases such as psoriasis or SLE.

## 1. Introduction

Toll-like receptors (TLRs) are the most upstream membrane-bound pathogen sensors in the innate arm of the vertebrate immune system [1]. These receptors are the mammalian counterpart of the *Drosophila* Toll protein, which plays an important role during dorso-ventral partitioning of the embryo and confers immune resistance in the fly [2,3]. TLRs are usually expressed on the membranes of professional immune cells, such as dendritic cells (DCs), macrophages, natural killer cells, and B and T lymphocytes [4]. These glycoproteins have an extracellular leucine-rich repeat (LRR) domain, a single transmembrane domain, and an intracellular Toll/interleukin-1 receptor (TIR) domain. To date, 10 functional members of the TLR superfamily have been identified in humans, of which TLRs 1, 2, 4, 5, 6, and 10 are found on the cell membrane, while TLRs 3, 7, 8, and 9 are localized to the endosomal membrane. Uniquely, TLR4 can function on the plasma membrane as well as on the endosomal membrane following endocytosis [5]. TLRs undergo homo- or heterodimerization after recognizing pathogen-associated molecular patterns (PAMPs) or damage-associated molecular patterns (DAMPs). Once activated, they trigger a complex signal transduction cascade that culminates in the production of proinflammatory cytokines and antiviral interferons (IFNs) [6]. These pattern recognition receptors recognize a wide range of PAMPs/DAMPs; for instance, triacyl lipoproteins (e.g., Pam_3_CSK_4_) are recognized by TLR1/2 [7], diacyl lipoproteins (e.g., Pam_2_CSK_4_) by TLR2/6 [8], lipopolysaccharide (LPS) by TLR4 [9], bacterial flagellin by TLR5 [10], viral double-stranded RNA (dsRNA) by TLR3 [11], viral single-stranded RNA (ssRNA) by TLR7 and TLR8 [12,13], and unmethylated CpG-containing oligodeoxynucleotide (ODN) by TLR9 [14]. To date, the natural agonist of TLR10 is unknown; however, its expression has been recorded in response to influenza virus infection [15]. While all TLRs undergo agonist-mediated homo/heterodimerization, TLR8 and TLR9 reportedly exist as preformed loose homodimers that become stabilized after agonist binding in the endosomal compartment [16].

Activated endosomal TLRs direct the translocation of transcription factors, IFN-regulatory factor 3 (IRF3; in case of TLR3), or nuclear factor kappa-light-chain-enhancer of activated B cells (NF-κB) into the nucleus, facilitating the expression of proinflammatory cytokines, such as tumor necrosis factor-α (TNF-α) and type I IFN [17]. TLRs 7, 8, and 9 belong to a category of TLR that recognize single-stranded nucleic acids of viruses, bacteria, or host origin and initiate the process of a sustained adaptive immune response [18]. TLR7 and TLR8 are homologous in terms of structure and function, recognizing ssRNAs from viruses, notably the influenza A virus, human immunodeficiency virus, and Dengue virus [19]. Despite the protective immune response of endosomal TLRs against invading pathogenic microorganisms, inappropriate engagement of TLR7/8 by host ssRNAs, such as microRNA or small interfering RNA released from dead/dying cells, propagates the pathogenesis of autoimmune diseases, namely psoriasis, systemic lupus erythematosus (SLE), and rheumatoid arthritis (RA) [20,21]. Owing to the clinical significance of endosomal TLRs, considerable efforts are being put forward to discover novel small molecule modulators that can act as therapeutic agents [22,23,24,25]. TLR modulators are attractive drug candidates, as evidenced by an increased number of anti-inflammatory compounds under consistent pharmaceutical research and development [26,27]. On the one hand, TLR agonists are used as immune response modifiers to treat genital warts, superficial basal cell carcinoma, and actinic keratosis [28,29], while antagonists are envisioned as therapeutic agents for treating autoimmune diseases, such as RA [30,31], SLE, and psoriasis [32,33].

Quantitative structure–activity relationship (QSAR) modeling is an established computational method increasingly used in the field of rational drug design to find novel compounds with improved bioactivities [34]. In the present study, we employed several online resources, including PubChem (https://pubchem.ncbi.nlm.nih.gov/), OCHEM (https://ochem.eu/home/show.do), and Chembench (https://chembench.mml.unc.edu/) [35] to screen a chemical database of ~8,000,000 compounds based on the QSAR modeling technique. Among the best predicted hits, a low molecular weight chemical compound named TLR antagonistic compound 5 (TAC5; 2-amino-3-benzyloxypyridine) and its synthetic derivatives [TAC5-a; 3-((4-aminobenzyl)oxy)pyridin-2-amine, TAC5-c; 3-(benzyloxy)-N-phenylpyridin-2-amine, TAC5-d; 3-(2-ethoxy-1-phenylethoxy)pyridine-2-amine, and TAC5-e; 3-(2-(2-aminoethoxy)-1-phenylethoxy)pyridin-2-amine] inhibited NF-κB-mediated expression of TNF-α/interleukin-6 (IL-6) triggered by TLR3/7/8/9 in both murine macrophage (RAW 264.7) and human monocytic cell lines (THP-1) with negligible cytotoxicity. In vivo, TAC5-a significantly ameliorated psoriasis disease symptoms in C57BL/6 mice as well as downregulated SLE disease markers in lupus-prone MRL/lpr mice. Together, our results demonstrate that the TAC5 series of compounds possess compelling therapeutic potential as prospective anti-inflammatory agents.

## 2. Experimental Section

### 2.1. Ligand Dataset for QSAR Modeling and Virtual Screening

Supplementary Table S1 shows the ligands and their sources, which were used to construct the QSAR models using the Chembench server [35]. The structural coordinates of the ligands were generated using MOE [36], under the guidelines presented by Walker and colleagues [34]. The molecules were optimized through energy minimization until a root mean square gradient of 0.05. After removing duplicate chemical structures, 234 ligands were stored in a database and were categorized into 128 active and 106 inactive compounds. The chemical properties of molecules were defined using descriptors of both Chembench and MOE. A multiconformational library of about 8,000,000 compounds was prepared using ligands from Chembridge (ChemBridge Corp., San Diego, CA, USA), the Traditional Chinese Medicine Taiwan database [37], MOE commercial database, and ZINC drug-like database [38]. A total of 2000 structures with 75% similarity to the dataset molecules were filtered out using a Scientific Vector Language (SVL) script. Virtual screening of the resultant 2000 compounds using different QSAR models suggested several hits, of which 50 compounds were found to be the consensus. The top 13 compounds were procured for experimental validation based on their purchasing availability (Supplementary Table S2).

### 2.2. Cell Culture and Reagents

Murine macrophages (RAW 264.7 cells) (ATCC, Manassas, VA, USA) were cultured in Dulbecco’s modified Eagle’s medium (DMEM; Thermo Fisher Scientific, Inc, Waltham, MA, USA) supplemented with 1% penicillin/streptomycin antibiotic mixture and 10% foetal bovine serum (FBS) (Thermo Fisher Scientific, Inc., Waltham, MA, USA). THP1-derived macrophage cells (kindly gifted by Dr. Chang-Hee Suh, Ajou University School of Medicine, Suwon, Korea) were cultured in RPMI 1640 medium containing 1% penicillin/streptomycin and 10% FBS (Thermo Fisher Scientific, Inc., Waltham, MA, USA), and then differentiated into macrophages with 80 nM phorbol 12-myristate 13-acetate (PMA; Sigma-Aldrich Co., St. Louis, MO, USA) for 24 h. The cells were incubated in a humidified atmosphere of 5% CO_2_ at 37 °C and the medium was changed after incubation overnight. Cells were treated with different TLR ligands. LPS (*Escherichia coli* 0111:B4) was purchased from Sigma-Aldrich Co. Pam_3_CSK_4_, FSL-1, poly(I:C), R848, CL075, imiquimod (IMQ; R837), TL8-506, and CpG ODN were all purchased from InvivoGen, San Diego, CA, USA. The compounds were dissolved in DMSO (Sigma-Aldrich Co., St. Louis, MO, USA) in brown tubes and stored at a concentration of 10 mM.

### 2.3. Cell Viability Assay

Cell viability was measured colorimetrically using the MTT solution (Sigma-Aldrich Co., St. Louis, MO, USA) diluted in phosphate-buffered saline (PBS). RAW 264.7 cells were seeded at a density of 2 × 10^5^ cells/well and treated with different concentrations of the small molecules for 24 h. The following day, the medium was removed and replaced with a diluted MTT solution (final concentration: 500 µg/mL) (100 µL/well). The cells were incubated for 3 h at 37 °C, after which the MTT solution was removed and a DMSO solution (100 µL/well; Sigma-Aldrich Co., St. Louis, MO, USA) was added. After 30 min, the absorbance at 540 nm was measured by a spectrophotometer (Molecular Devices, Inc., San Jose, CA, USA).

### 2.4. Detection of TNF-α Secretion

RAW 264.7 cells and THP1-derived macrophages were seeded in a 96-well plate (BD Biosciences, San Jose, CA, USA), at a density of 2 × 10^5^ cells/well and grown overnight. After 24 h of treatment, the secretion level of TNF-α was measured from culture supernatants using the Mouse TNF-α ELISA Ready-SET-Go! kit (eBioscience, San Diego, CA, USA) or the Human TNF-α ELISA MAX Set Deluxe (BioLegend, San Diego, CA, USA) kit. All experiments were conducted according to the manufacturer’s instructions. The absorbance was measured at 450 nm on a microplate reader spectrophotometer (Molecular Devices, Inc., San Jose, CA, USA), and the data were analyzed against a standard curve using SoftMax Pro 5.3 software (Molecular Devices, Inc., San Jose, CA, USA).

### 2.5. Measurement of Protein Expression Levels Using Western Blotting

After treatment, the proteins were isolated by resuspending cells in M-PER Mammalian Protein Extraction Reagent (Thermo Fisher Scientific, Inc., Waltham, MA, USA) or NE-PER lysate (Thermo Fisher Scientific, Inc., Waltham, MA, USA) with protease and phosphatase inhibitor cocktails (Thermo Fisher Scientific, Inc., Waltham, MA, USA), incubated for 10 min at 4 °C, and centrifuged at 16,000× *g* for 10 min to remove cell debris. Extracts were loaded onto 10% polyacrylamide gels and transferred to Hybond-ECL membranes (Amersham, Pollards Wood, UK). Membranes were blocked with 5% skim milk in PBS containing 0.05% Tween 20 (PBST). The membranes were then incubated with specific primary antibodies against histone deacetylase 1 (HDAC1; Merck Millipore, Billerica, MA, USA), NF-κB (p65), IκBα, p-ERK, ERK, p-JNK, JNK, p-p38, p38, activating transcription factor 3 (ATF3), β-actin (Santa Cruz Biotechnology, Inc., Dallas, TX, USA), or p-p65 (Cell Signaling Technology, Inc., Danvers, MA, USA) diluted with PBST at 4 °C with gentle shaking overnight. After several washes, the membranes were incubated with an anti-mouse or anti-rabbit immunoglobulin G peroxidase-conjugated antibody (Thermo Fisher Scientific, Inc., Waltham, MA, USA) diluted in PBST (1:1000) for 2 h. After the membranes were washed several times with PBST, the detection was carried out using Pierce ECL western blotting substrate (Thermo Fisher Scientific, Inc., Waltham, MA, USA) and by exposing the blots to a LAS-1000 system (Fuji, Tokyo, Japan).

### 2.6. Confocal Microscopy Analysis

RAW 264.7 cells were fixed in 3.7% formaldehyde (Sigma-Aldrich, Co., St. Louis, MO, USA) for 5 min and permeabilized with 0.2% Triton X-100 for 5 min. The fixed cells were blocked with 5% FBS (Thermo Fisher Scientific Inc., Waltham, MA, USA) in PBS for 30 min and then incubated with primary antibodies (1:1000, 1 h) targeting p-p65 (Cell Signaling Technology, Inc., Danvers, MA, USA). Next, the primary antibody bound-cells were incubated with secondary antibodies (1:1000, 1 h) conjugated with Alexa Fluor 546 (Invitrogen, Carlsbad, CA, USA). Nuclei were stained with Hoechst 33,258 (5 mM; Sigma-Aldrich, Co., St. Louis, MO, USA) for 10 min. Stained cells were visualized using confocal microscopy (LSM-700; Carl Zeiss Microimaging, Jena, Germany) and analyzed by Zen 2009 software (https://www.zeiss.com).

### 2.7. In Vivo Study Using a Mouse Model of Psoriasis

To determine the therapeutic effects of TAC5-a in vivo, C57BL/6 mice aged 6–7 weeks old were purchased from Orient Bio, Inc. (Seongnam, South Korea). The mice were housed under specific pathogen-free conditions and provided with a standard laboratory diet and fed ad libitum. All animal experiments were approved by the Institutional Animal Care and Use Committee (IACUC) at the Ajou University (Approval No. 2017-0002). Psoriasis symptoms were generated by topically applying 42 mg IMQ (Aldara) cream (5%; 3 M Pharmaceutical Co., Maplewood, MN, USA) for six consecutive days. Aldara was not applied to the control group. Mice were administered with daily doses of TAC5-a (50, 100, or 200 nM; i.e., 10.8, 21.6, or 43.2 mg/mL, respectively) or PBS as a control, starting at one day before applying the Aldara cream. MTX (20 mg/mL; M9929, Sigma-Aldrich, Co., St. Louis, MO, USA) was also administered as the positive control starting at one day before applying the Aldara cream. After seven days, mice were sacrificed under respiratory anesthetic, and skin lesions, serum samples, and spleens were collected for analysis. Histological analysis & immunochemical staining were performed on the skin samples from back lesions of the mice. The samples were fixed with 4% paraformaldehyde solution, embedded in paraffin, and sectioned to 7-μm onto glass slides. The sections were then stained with hematoxylin and eosin (H&E) to evaluate the thickness of the dermis and epidermis. The thickness of the skin was measured with the Leica DMi8 fluorescence microscope using Leica LAS X Hardware Configurator (Leica Microsystems GmbH, Wetzlar, Germany). IMQ-mediated skin inflammation was evaluated by immunohistochemistry (IHC) using primary antibodies recognizing CD68 (ab31630, dilution of 1:200) as the macrophage marker.

To score the inflammation severity of the back skin, an objective scoring system was developed based on the clinical Psoriasis Area and Severity Index (PASI). Erythema, scaling, wrinkle, and thickening were scored independently from 0 to 4 as follows: 0, none; 1, slight; 2, moderate; 3, marked; and 4, very marked. The level of erythema, scaling, and thickness was scored under the careful observation of experienced researchers. The cumulative score (erythema plus scaling plus thickening, scale 0–12) indicated the severity of the psoriasis symptoms.

### 2.8. In Vivo Study Using a Mouse Model of SLE

Five female wild-type (C57BL/6) and lupus-prone mice (MRL/lpr), each initially weighing 18–20 g and 38–40 g, respectively, were purchased from The Jackson Laboratory (Bar Harbor, ME, USA). All animal procedures were reviewed and approved by the animal ethics committee of Ajou University Medical Center (Approval No. 2017-0022). Mice were allowed to acclimatize for one week before the experiment began. They were bred and maintained under pathogen-free conditions according to the guidelines issued by the animal facility of Ajou University School of Medicine. 

The mice from vehicle groups received an intraperitoneal (IP) injection of 1% DMSO and the mice from the TAC5-a treatment groups received an IP injection of TAC5-a (10 nmol/g/day, dissolved in 1% DMSO) for 11 days. During the treatment period, mice were weighed daily. At the end of experiments, mice were sacrificed, and blood, urine, and tissues were collected. After a 1 h incubation, blood samples were collected into serum separation tubes and centrifuged at 3000 rpm for 10 min at 20 °C and then serum samples were stored at −80 °C. Collected urine samples were immediately stored at −80 °C. Tissues were rapidly removed, thoroughly washed with PBS, and immersed in RNA stabilization solution (QIAGEN Sciences, Maryland, MD, USA).

The concentration of dsDNA antibodies and ANAs in mice serum was analyzed by ELISA. Specifically, mouse urinary albumin levels were analyzed with a mouse albumin ELISA kit (41-ALBMS-E01, Alpco Diagnostics, Salem, NH, USA) according to the manufacturer’s protocol. Three independent measurements per sample were performed.

### 2.9. Quantitative Real-Time PCR (qRT-PCR) 

qRT-PCR analysis was performed to identify the expression levels of specific genes in the lymph node, spleen, and kidney. For this task, total RNA was isolated from the lymph node, spleen, and kidney of 16-week old mice using TRI Reagent (Sigma-Aldrich, Co., St. Louis, MO, USA). RNA was subjected to qRT-PCR using SYBR green-based (Enzo Life Sciences, Inc., Farmingdale, NY, USA). The following primers for the marker genes were used: *mTlr7*, 5′-ATG TGG ACA CGG AAG AGA CAA-3′ and 5′-GGT AAG GGT AAG ATT GGT G-3′; *mTlr9*, 5′- ATG GTT CTC CGT CGA AGG ACT-3′ and 5′-GAG GCT TCA GCT CAC AGG G-3′; *mIl-6*, 5′-GAG GAT ACC ACT CCC AAC AGA CC-3′ and 5′-AAG TGC ATC ATC GTT GTT CAT ACA-3′; mMyd88, 5′-CAC CTG TGT CTG GTC CAT TG-3′ and 5′-CTG TTG GAC ACC TGG AGA CA-3′; *mIl-17*, 5′-GCT CCA GAA GGC CCT CAG A-3′ and 5′-AGC TTT CCC TCC GCA TTG A-3′; *mGapdh*, 5′-CTC AAC ACG GGA AAC CTC AC-3′ and 5′-CGC TCC ACC AAC TAA GAA CG-3′ (Bioneer, Daejeon, Korea). The quantified individual RNA expression levels were normalized to *Gapadh* and depicted as relative RNA expression levels with the corresponding wild-type mice.

### 2.10. Evaluation of TAC5 Binding to TLR7 Using the Surface Plasmon Resonance (SPR) Assay

SPR experiments were conducted on a Reichert SR7500DC machine (Reichert Technologies Depew, New York, NY, USA) using the carboxymethyldextran hydrogel (CMDH) sensor chip. The recombinant mouse TLR7 protein (R&D systems) was immobilized onto the sensor chip at a concentration of 5 µg/mL using 10 mM sodium acetate solution at pH 4.0. PBS containing 5% of DMSO served as a running buffer and 20 mM NaOH solution was used for regeneration. Different concentrations of TAC5 and IMQ ranging from 0 to 400 μM were injected into the chip to evaluate their binding to TLR7; running buffer was injected into the empty channel and was used as a reference. For the evaluation of the competitive binding between the ligand for TLR7 (Imiquimod; IMQ) and TAC5, IMQ was injected at the concentration of 100 μM, in the presence of different concentrations of TAC5 ranging from 0 to 400 μM. The experiments were conducted in duplicate with freshly prepared reagents, and data analysis was performed using the Scrubber2 software. 

### 2.11. Molecular Docking

The crystal structures of monkey TLR7 and human TLR8 were downloaded from Protein Data Bank (PDB) using the PDB IDs 5GMH [39] and 3W3J [16], respectively. The model of human TLR7 was constructed through homology modeling using 5GMH as a template in the SWISS-MODEL server [40]. The two structures were optimized by energy minimization in the MOE software. The chemical structure of TAC5-a was docked with the optimized models of TLR7 and TLR8 using MOE. The ligand-binding site was defined around the ligands present in the respective crystal structures. The MMFF94x forcefield and London dG scoring function were used for the docking calculation. A total of 30 confirmations were generated in each docking run and the best one was chosen according to the highest binding affinity score (i.e., the S score). The docked complexes were energy minimized and subjected to molecular dynamics simulations using the GROMACS 5.1.4 program [41], as described below.

### 2.12. Molecular Dynamics Simulations

The docked complexes of TLR7-TAC5-a and TLR8-TAC5-a were processed using GROMOS96-54A7 forcefield and solvated inside cubic boxes, followed by solvating with simple point charge water molecules. The Gromacs-compatible ligand topology was obtained from the PRODRG server [42]. The simulation systems were electroneutralized by adding an appropriate amount of counterions (Na^+^/Cl^–^). Steepest descent energy minimizations on both the systems were performed until a termination gradient of 0.001 kJ mol^−1^ nm^−1^ was reached. The minimized structures were then equilibrated for temperature and pressure using the Nose–Hoover and Parrinello–Rahman methods, respectively, up to 100 ps. During this process, positional restraints were applied to the backbone heavy atoms. The equilibrated systems were simulated for 100 ns using the NPT ensemble (constant pressure, constant temperature) without backbone restraints. The van der Waals and electrostatic interactions were estimated by applying a 12 Å distance cut-off. Long-range electrostatic interactions were calculated with the particle mesh Ewald algorithm. The simulations were performed under periodic boundary conditions and the LINCS algorithm was employed to constrain all bonds involving hydrogen atoms. Structural coordinates were saved at 2-ps time intervals. Structure visualizations were accomplished using MOE or VMD [43] programs.

### 2.13. Statistical Analysis

All data presented were obtained from independent experiments with one-way analysis of variance using SigmaPlot software version 12.0 (Systat Software Inc., San Jose, CA, USA). Data analyses of the in vivo studies were performed using SPSS (version 23.0; Chicago, IL, USA) installed on a Windows operating system. The data are shown as means ± standard deviation (SD) or median and interquartile range, as appropriate. The SLE data showing the differences in antibody/protein levels were determined using the Wilcoxon Rank Sum test (which is numerically equivalent to the Mann–Whitney *U* test). *p* values < 0.05 and < 0.01 were regarded as statistically significant. 

## 3. Results

### 3.1. Identification of Probable Inhibitors of Endosomal TLRs

The initial lead compound of this study was identified by employing a QSAR modeling approach, as described by Tropsha [34]. The total computational workflow is illustrated in Supplementary Figure S1. The ligand dataset was split into 128 active and 106 inactive molecules based on the compounds’ activity data reported in the respective literature (Supplementary Table S1). The QSAR models were constructed using three different algorithms: k-Nearest Neighbor (k-NN), Random Forest (RF), and Support Vector Machine (SVM). The chemical characteristics of each molecule were represented by either DragonX-H or MOE descriptors. The three QSAR methods (k-NN, RF, and SVM) resulted in predictive models that satisfied the statistical threshold Q^2^ or the correct classification rate (CCR) of 0.7 (Table 1), which are the acceptable criteria for a reasonable prediction. 

**Figure 1:**
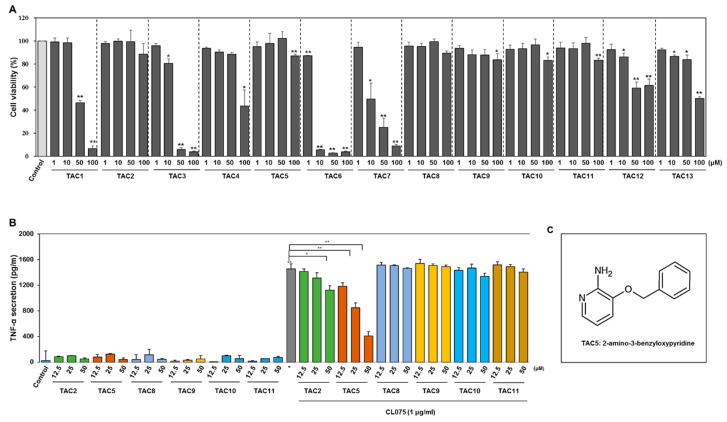
Cell viability measurement and TNF-α-inhibitory profile of TAC5. (**A**) RAW 264.7 cells were treated with selected compounds at different concentrations (1, 10, 50, or 100 µM) for 24 h. Cell viability was measured by a 3-(4,5-dimethylthiazol-2-yl)-2,5-diphenyltetrazolium bromide (MTT) assay. The percentage of viable cells as compared to control is presented in a histogram. All experiments were independently conducted three times and mean ± SEM of the independent experiments was calculated by a two-tailed paired Student’s *t*-test (* *p* < 0.05, ** *p* < 0.01). (**B**) RAW 264.7 cells were treated with the compounds TAC2, TAC5, and from TAC8 to TAC11 at concentrations of 12.5, 25, and 50 µM in the presence and/or absence of CL075 for 24 h. The secreted levels of TNF-α were measured in the cell culture supernatant by a TNF-α enzyme-linked immunosorbent assay (ELISA) kit. (**C**) A two-dimensional representation of the chemical structure of TAC5. All experiments were independently conducted five times and the mean ± SEM of the independent experiments was calculated by a two-tailed paired Student’s *t*-test (* *p* < 0.05, ** *p* < 0.01).

Virtual screening was performed against a chemical compound library of 2000 molecules that were filtered using a fingerprint-based method from a multiconformational library of ~8,000,000 molecules. The top 13 high-ranking consensus hits were selected for experimental validation of activity (Supplementary Table S2). To identify the most potent candidate among the 13 selected compounds, RAW 264.7 cells were incubated with 1, 10, 50, or 100 µM of the compounds. After 24 h, a 3-(4,5-dimethylthiazol-2-yl)-2,5-diphenyltetrazolium bromide (MTT) assay was performed (Figure 1A), showing that TAC1, TAC3, TAC4, TAC6, TAC7, TAC12, and TAC13 were cytotoxic. Therefore, TAC2, TAC5, and from TAC8 to TAC11 were considered for further experiments to assess their activity using enzyme-linked immunosorbent assays (ELISA). Among the selected five compounds, none showed agonistic activity, while TAC2 and TAC5 inhibited CL075 (TLR7/8 agonist)-induced TNF-α secretion (Figure 1B). Furthermore, TAC5 showed a better inhibitory effect compared to that of TAC2 in a dose-dependent manner. The chemical structure of TAC5 is shown in Figure 1C.

### 3.2. Effect of TAC5 on CL075-Induced TLR7 and TLR8 Signaling

**Figure 2:**
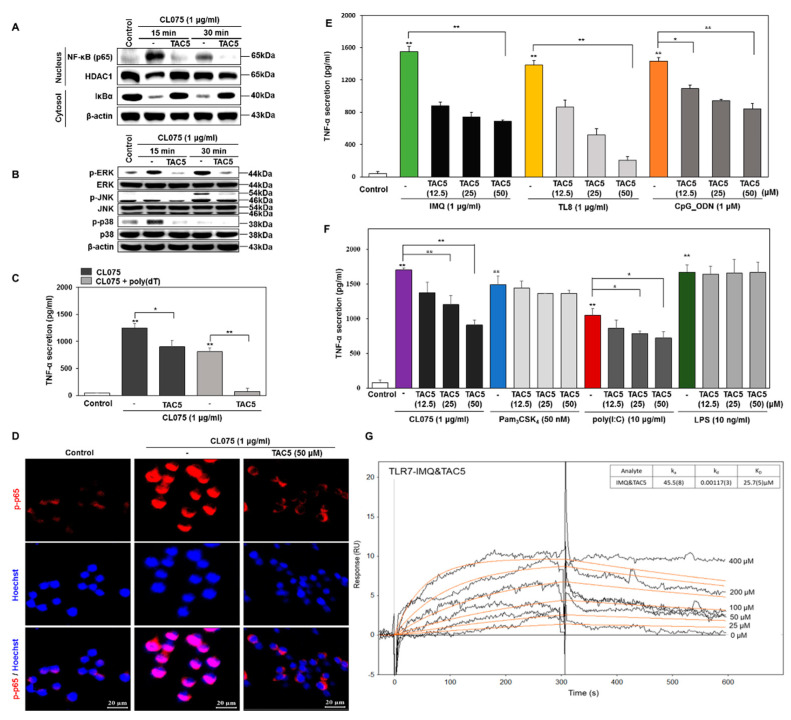
TAC5 inhibits the CL075-induced proinflammatory response. RAW 264.7 cells were treated with 50 µM of TAC5 for 1 h, followed by treatment with CL075 (1 µg/mL) for 15 and 30 min, respectively. (**A**) Nuclear factor kappa-light-chain-enhancer of activated B cells (NF-κB) activation (NF-κB translocation into the nucleus and degradation of IκBα in the cytosol) was measured by western blotting. Histone deacetylase-1 was used as a nucleus loading control. β-actin was used as a cytosol loading control. (**B**) The expression levels of mitogen-activated protein kinases (MAPKs) were evaluated by western blot analysis using whole protein extracts, with inactive MAPKs used as controls (β-actin was used as a loading control). (**C**) Inhibition of TNF-α secretion by TAC5 in the presence of CL075 and a combination of CL075 and poly(dT). (**D**) Confocal microscopy image of the phosphorylated NF-κB (p-p65) expression level. The red staining corresponds to phosphorylated p65 subunit of NF-κB (p-p65) and blue staining (Hoechst33258) indicates nuclei (the scale bar is 20 µm in width). (**E**,**F**) The expression level of TNF-α was measured by ELISA when the TAC5 was co-treated with (**E**) imiquimod (TLR7), TL8-506 (TLR8), CpG ODN (TLR9) (F) Pam_3_CSK_4_ (TLR1/2), poly(I:C) (TLR3), LPS (TLR4), or CL075 (TLR7/8) agonists in RAW 264.7 cells. (**G**) Surface plasmon resonance (SPR) sensorgram illustrating the competitive binding of TAC5 to TLR7 in the presence of imiquimod. The data shown represent five independent experiments, and the bars represent means ± SEM (* *p* < 0.05, ** *p* < 0.01).

Our cell-based assays suggested that TAC5, among other TACs, had a prominent antagonistic effect on the CL075-induced activation of TLR7 and TLR8 in mouse macrophages (RAW 264.7 cells). Western blot analysis revealed that TAC5 inhibited CL075-induced NF-κB activation and IκBα degradation in RAW 264.7 cells (Figure 2A). TLRs are known to induce the activation of mitogen-activated protein kinases (MAPKs), c-Jun N-terminal kinase (JNK), extracellular signal-regulated kinase (ERK), and p38 MAPK that regulate various cell functions including proliferation and apoptosis. We found that TAC5 suppressed the CL075-induced activation of MAPKs (i.e., the phosphorylation of JNK, ERK, and p38) (Figure 2B). Next, RAW 264.7 cells were incubated either with CL075 or with a combination of CL075 and poly(dT), which reportedly elevates TLR8-mediated signaling [44,45]. Measurements of the TNF-α secretion level revealed that TAC5 showed a slightly improved inhibitory effect on the poly(dT) treated cells as compared to the cells treated with only CL075 (Figure 2C). CL075-induced NF-κB activation was also studied through confocal microscopy using immunofluorescent staining in RAW 264.7 cells. TAC5 significantly decreased the expression of phosphorylated p65 (p-p65), indicating the inactivation of NF-κB (Figure 2D). 

To further evaluate the inhibitory effect of the selected compound on other TLRs, RAW 264.7 cells were co-treated with TAC5 in the presence of TLRs ligands: imiquimod (IMQ; TLR7), TL8-506 (TLR8), poly(I:C) (TLR3), CpG ODN (TLR9), CL075 (TLR7/8), Pam_3_CSK_4_ (TLR1/2 agonist) or LPS (TLR4 agonist). Interestingly, our data indicated that TAC5 has a significant inhibitory effect on TLR7/8/9-meditated TNF-α secretion and a moderate effect on poly(I:C)-induced TLR3-mediated TNF-α production (Figure 2E,F). On the other hand, TNF-α secretion was not affected in cells treated with Pam_3_CSK_4_ (TLR1/2 agonist) or LPS (TLR4 agonist). Furthermore, to evaluate the competitive binding of TAC5 and IMQ to TLR7, we conducted an SPR assay using the recombinant mouse TLR7 protein. Initially, the binding affinity of TAC5 and IMQ to the TLR7 was conducted. The sensorgram for TAC5 (Supplementary Figure S3A) showed that the compound binds slowly to TLR7 and does not dissociate quickly. The calculated association (ka) and dissociation (kd) rate constants were found to be 34.3 M^−1^ s^−1^ and 6.8(1) × 10^−4^ s^−1^, respectively), as compared to IMQ (Supplementary Figure S3B), which presented a relatively lower affinity to the bound TLR7 (ka = 2.2(1) × 10^2^ M^−1^ s^−1^, kd = 7.8(3) × 10^−2^ s^−1^). Furthermore, the sensorgram of the competitive binding of different concentration of TAC5 and IMQ (100 µM) to TLR7 showed a dose-dependent binding of the compound and a slow association/dissociation rate was observed with a binding affinity similar to TAC5. This suggests that TAC5 presents a strong and competitive binding to TLR7 despite the presence of IMQ (Figure 2G).

### 3.3. TNF-α Inhibition Profile of a 3-Amino-Derivative of TAC5 (TAC5-a)

**Figure 3:**
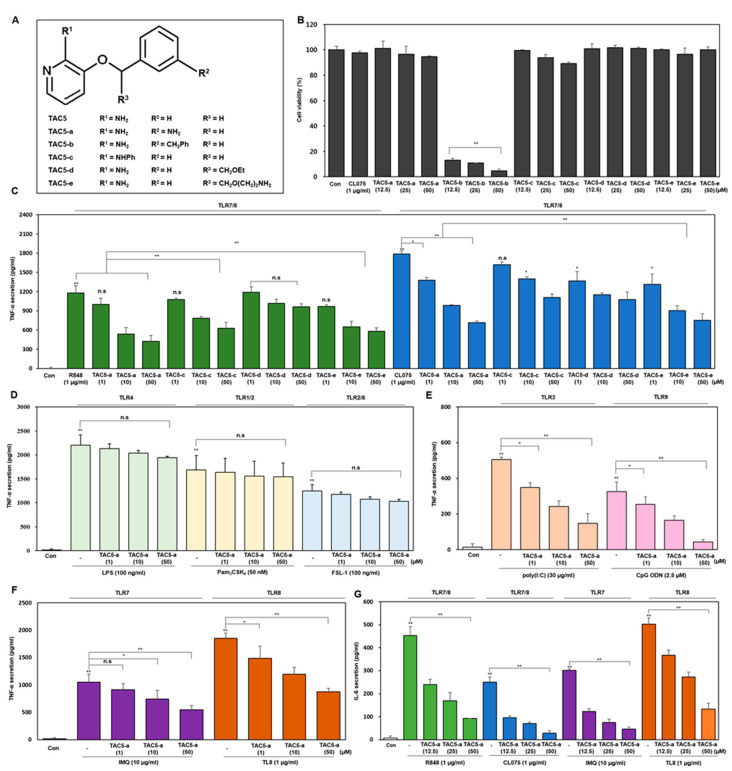
Inhibitory effect of TAC5-a on the TLR signaling pathways. (**A**) The chemical structure of TAC5 and its derivatives represented by different R groups. (**B**) RAW 264.7 cells were treated with various concentrations of compounds for 24 h and the cell viability was measured using an MTT assay. (**C**) The inhibitory effects of TAC5-a, TAC5-c, TAC5-d, and TAC5-e on the TNF-α secretion level in THP1 derived macrophage cells under the influence of different TLR7/8 ligands. R848 and CL075 were used to activate TLR7/8, imiquimod (IMQ) to activate TLR7, and TL8-506 to activate TLR8, specifically. (**D**–**F**) The inhibitory effect of TAC5-a on the TNF-α secretion level in THP1-derived macrophage cells was evaluated following the treatment of cells with different TLR ligands (**D**) Lipopolysaccharide was used to activate TLR4, Pam_3_CSK_4_ to activate TLR1/2, and FSL-1 to activate TLR2/6). (**E**) poly(I:C) was used to activate TLR3 and CpG ODN to activate TLR9. (**F**) R848 and CL075 were used to activate TLR7/8, IMQ to activate TLR7, and TL8-506 to activate TLR8. (**G**) The inhibitory effects of TAC5-a on the IL-6 secretion in RAW264.7 cells stimulated with agonists of TLR7/8 (R848 and CL075), TLR7 (IMQ), or TLR8 (TL8-506). All data shown represent the results of five independently conducted experiments; the mean ± SEM of the independent experiments were calculated and used in the two-tailed paired Student’s *t*-test (* *p* < 0.05, ** *p* < 0.01).

TAC5 has a simple structure of a 2-aminopyridine moiety connected by a methoxy group to a benzene ring (Figure 1C). In an attempt to improve the activity of TAC5, we performed five modifications to its structure, resulting in the derivatives TAC5-a (3-((4-aminobenzyl)oxy)pyridin-2-amine), TAC5-b (3-((3-benzylbenzyl)oxy)pyridin-2-amine), TAC5-c (3-(benzyloxy)-N-phenylpyridin-2-amine), TAC5-d (3-(2-ethoxy-1-phenylethoxy)pyridine-2-amine), and TAC5-e (3-(2-(2-aminoethoxy)-1-phenylethoxy)pyridin-2-amine) (Figure 3A and Supplementary Figure S2). Based on the X-ray crystal structures of TLR7 and TLR8, it is clear that the ligand-binding site comprises a large number of nonpolar amino acids and very few polar (threonine/tyrosine) or acidic (aspartate) amino acids. Therefore, the rationale for the analogues prepared was to add aromatic/aliphatic or positively charged moieties to the selected R-groups of TAC5 structure in order to increase its affinity towards the receptor. The TAC5-derivatives were tested for their cytotoxicity by treating RAW 264.7 cells with concentrations between 12.5 and 50 µM. This preliminary investigation revealed that TAC5-b significantly decreased cell viability, while TAC5-c was slightly cytotoxic at 50 µM (Figure 3B). On the other hand, the rest of the compounds had no cytotoxic effect on the cells. TAC5-a, TAC5-c, TAC5-d, and TAC5-e inhibited TLR7/8-mediated TNF-α expression in THP-1 cells (a human monocytic cell line) dose-dependently (Figure 3C). As TAC5-a showed a greater TNF-α inhibition among all derivatives, further experiments were conducted using this compound at concentrations between 1 and 50 µM. The inhibitory effect of TAC5-a on TLR-mediated signaling was confirmed by measuring the secretion of TNF-α through ELISA in THP1-derived macrophages. As expected, the compound did not block TLR4 (LPS)-, TLR1/2 (Pam_3_CSK_4_)-, and TLR2/6 [Pam_2_CGDPKHPKSF (FSL-1)]-mediated TNF-α secretion (Figure 3D). Further, to assess the inhibition of endosomal TLRs, cells were incubated with TAC5-a in the presence of either R848 or CL075 (TLR7/8), imiquimod (IMQ; TLR7), TL8-506 (TLR8), poly(I:C) (TLR3), or CpG ODN (TLR9) ligands (Figure 3E,F). Results showed that TAC5-a significantly suppressed TLR3/7/8/9-mediated TNF-α secretion in a dose-dependent manner. This suggests that TAC5-a is a potential and specific blocker of endosomal TLR activation. Additionally, TAC5-a and TAC5-c exerted dose-dependent inhibition of IL-6 expression in cells treated with either R848 (TLR7/8), CL075 (TLR7/8), IMQ (TLR7 specific), or TL8-506 (TLR8 specific) ligands (Figure 3G and Supplementary Figure S3B,C).

### 3.4. Effect of TAC5-a Treatment on Mouse Models of Psoriasis and SLE

**Figure 4:**
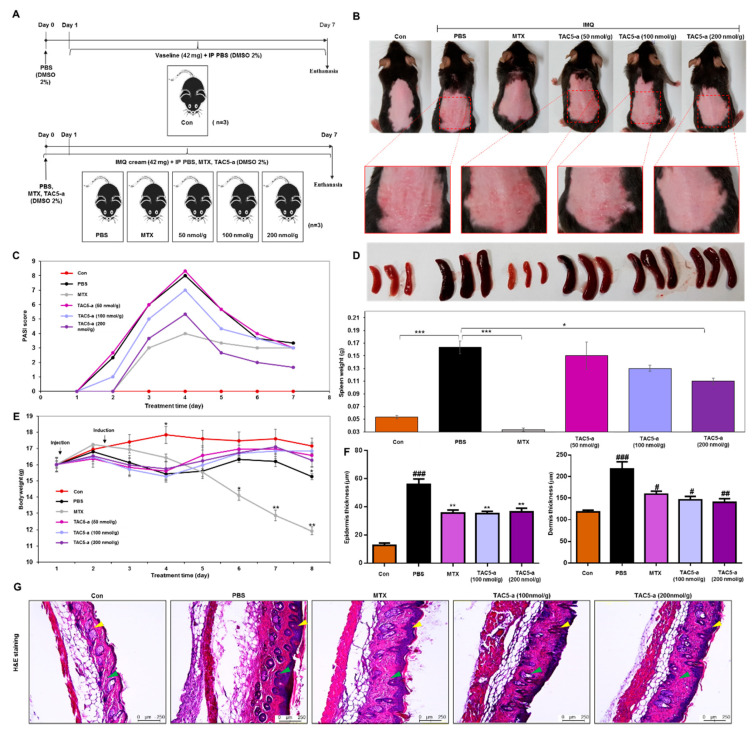
Effect of TAC5-a in a mouse model of psoriasis. (**A**) Summary of the experiment for evaluating the therapeutic effect of TAC5-a on a psoriasis mouse model. Psoriasis was induced by applying imiquimod (IMQ) cream (5%) on the shaved back skin of C57BL/6 male mice (*n* = 3). Mice were treated with daily doses of PBS as a control and 50, 100, and 200 nmol/g of TAC5-a intraperitoneally (IP). Methotrexate (MTX; 20 μg/g) was administered as a positive control from one day before applying IMQ cream as a pretreatment. (**B**) Photographs of shaved mouse back skin on day 4 to monitor the effect of TAC5-a on psoriasis condition. (**C**) Scoring of disease severity based on the clinical Psoriasis Area and Severity Index (PASI). Orange, dark blue, and green lines indicate the PASI index of mice treated with TAC5-a at 50, 100, and 200 nmol/g, respectively. (**D**) Effect of TAC5-a on spleen weight. (**E**) Body-weight dynamics of mice during the treatment regimen. (**F**) The thickness of the epidermis and dermis in TAC5-a and MTX (positive control) group. (**G**) The Histopathological changes of back skin lesions of each group. Skins were stained with hematoxylin and eosin to evaluate the thickness of the epidermis (yellow arrow) and dermis (green arrow) in IMQ-induced psoriasis-like mice. The skin thickness of each group was measured with the Leica DMi8 fluorescence microscope. Data represent mean ± SEM from five skin tissue of each group by two-tailed Student’s *t*-test, # *p* < 0.05, ## *p* < 0.01, ### *p* < 0.001, * *p* < 0.05, ** *p* < 0.01, *** *p* < 0.001.

The therapeutic efficacy of TAC5-a was evaluated in a mouse model of psoriasis and a mouse model of SLE. Psoriasis was induced in 6–7-week-old C57BL/6 mice by the topical application of 42 mg IMQ cream (5%) on shaved back skin for six consecutive days (Figure 4A). TAC5-a was injected daily at doses of 50, 100, and 200 nmol/g and the effect on disease severity was analyzed. A reversal of inflammation severity was observed on the back skin of the TAC5-a-treated mice (Figure 4B), although the effect was relatively weaker than the effect of the chemotherapeutic drug methotrexate (MTX). Scoring using Psoriasis Area and Severity Index (PASI) revealed that TAC5-a significantly reduced the PASI score from >8.0 on day 4 to a plateau at <4.0 after day 5 of the treatment (Figure 4C). Accordingly, TAC5-a treatment moderately prevented splenomegaly (Figure 4D), which was directly correlated to the body-weight dynamics of the treatment group (Figure 4E). Psoriasis is commonly marked by dilated blood vessels and thickening of the epidermis and the dermis due to the infiltration of immune cells, such as macrophages and T cells. Histopathological examination revealed that TAC5-a treatment reduced the epidermal plaques and decreased the skin thickness, which was induced through IMQ application (Figure 4F,G). 

**Figure 5:**
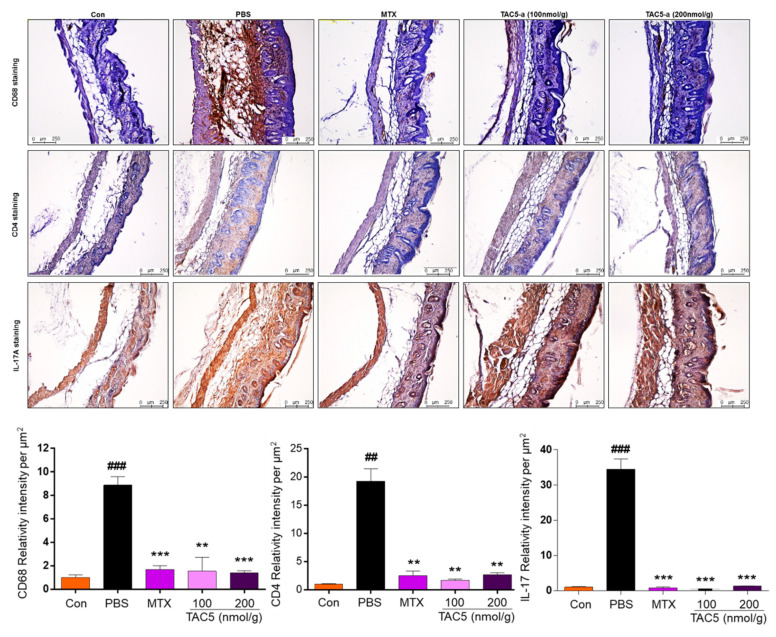
Immunohistochemical analysis of the back skin lesions of each group. The immunohistochemistry evaluation was performed by using primary antibodies recognizing CD68. TAC5-a effectively decreased the expression of CD68, CD4, and IL-17A (brown). Data represent mean ± SEM from five skin tissues of each group by two-tailed Student’s *t*-test, ## *p* < 0.01, ### *p* < 0.001, ** *p* < 0.01, *** *p* < 0.001.

Furthermore, the immunohistochemical analysis indicated a greater reduction of macrophage (CD68) and TH17 (CD4 and IL-17) cells infiltration in the dermis (Figure 5). This suggested that TAC5-a has the potential to relieve psoriatic diseases, such as acanthosis, parakeratosis, and hyperkeratosis. 

**Figure 6:**
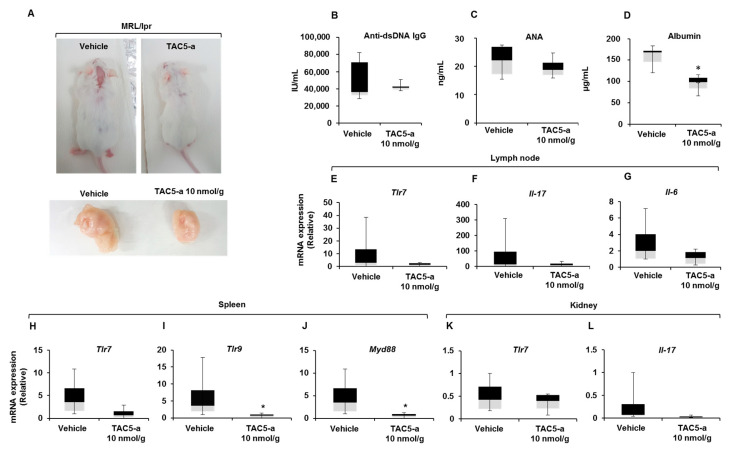
Effect of TAC5-a in a mouse model of systemic lupus erythematosus (SLE). (**A**) Effect of TAC5-a treatment in lupus-prone mice (MRL/lpr) having the SLE phenotype (*n* = 5). The enlargement of the lymph node was markedly reduced in TAC5-a-treated mice. (**B**) Amount of anti-dsDNA antibodies and (**C**) antinuclear antibody in serum, as determined by an ELISA. (**D**) The albumin content in urine, as determined by the ELISA. (**E**–**L**) The relative expression levels of specific genes in the lymph node, kidney, and spleen from MRL/lpr mice, determined by quantitative real-time PCR. The name of the analyzed gene is indicated in each panel. Data were normalized to *Gapdh*. The exact Wilcoxon Rank Sum test (which is numerically equivalent to the Mann–Whitney *U* test) was performed to compare the mean values in the two groups. * *p* < 0.05.

The therapeutic potential of TAC5-a against systemic autoimmunity was further studied using MRL/lpr mice by intraperitoneally injecting daily doses of 10 nmol/g of TAC5-a (dissolved in 1% dimethyl sulfoxide [DMSO]) for 11 days. The inhibitor affected mice lymphoproliferation; lymphoid expansion was significantly prevented in the TAC5-a-treated mice as compared to that in vehicle-treated mice (Figure 6A). The production of anti-dsDNA autoantibodies remained low (Figure 6B), and the serum antinuclear antibodies (ANAs) were decreased by treatment with the inhibitor (Figure 6C). The detection of certain proteins, albumin in particular, in the urine serves as the marker of renal pathology called glomerulonephritis (GN), which is caused by the inflammation of glomeruli. As shown in Figure 6D, the content of urine albumin was significantly reduced by TAC5-a treatment, exhibiting its therapeutic capability against GN manifestation. Next, we examined the effects of TAC5-a on the relative expression patterns of specific genes in the lymph node, spleen, and kidney at the transcription level. TAC5-a markedly reduced TLR7, IL-17, and IL-6 mRNA levels in the lymph node (Figure 6E–G); TLR7, TLR9, and MyD88 mRNA expressions in the spleen (Figure 6H–J), and TLR7 and IL-17 expressions in the kidney (Figure 6K,L). This implies that the prevention of the enlargement of the lymph node, as shown in Figure 6A, could be attributed to the downregulation of proinflammatory cytokines. 

### 3.5. Prediction of the Binding Modes of TAC5-a on TLR7/8

**Figure 7:**
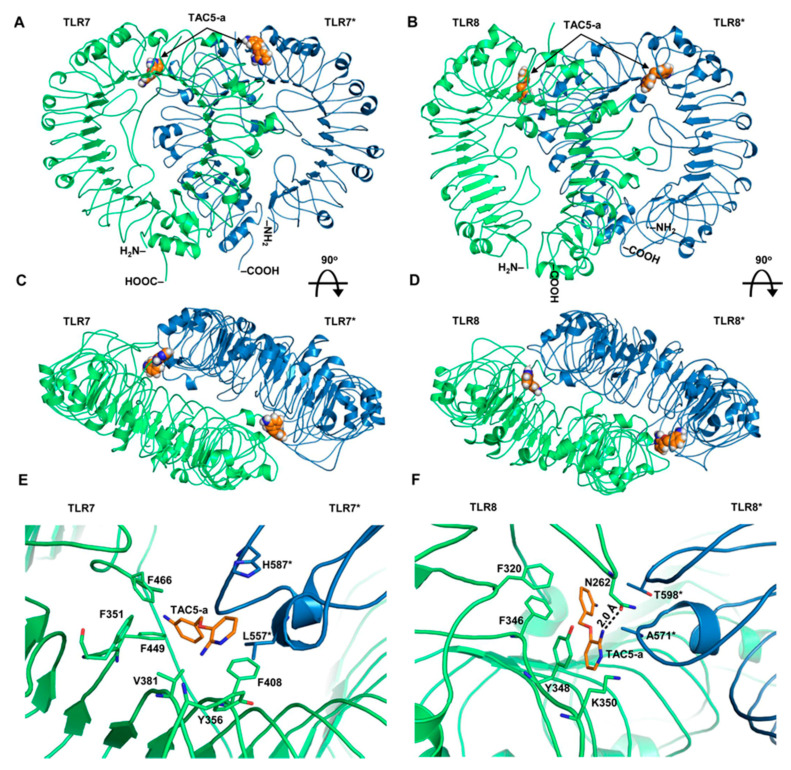
Structural models of TAC5-a bound to TLR7 and TLR8. (**A**) The overall structure of a homology model of human TLR7 having TAC5-a at the synthetic agonist binding cavity. (**B**) The structure of human TLR8 with TAC5-a bound to the agonist-binding site. (**C**) The top surface of TLR7 after 90° rotation of panel (**A**). (**D**) A 90°-rotated view of the panel (**B**). (**E**) Detailed intermolecular interaction of TAC5-a with the amino acids of TLR7. ‘*’ indicates chain B of the protein. (**F**) A close-up view of the intermolecular contacts between TAC5-a and TLR8 residues. ‘*’ indicates subunit B of the protein. The hydrogen bond (H-bond) is shown as a dashed line. The digit indicates the distance of the H-bond in Å. TAC5-a is shown as an orange stick. The chain As of both proteins are colored lime green and the chain Bs are sky blue.

After confirming the in vitro and in vivo efficacy of TAC5-a, we sought to predict its binding mode and interaction pattern with TLR7 and TLR8. As the agonist/antagonist binding locations on these receptors have been well defined by X-ray crystallography studies [16,39], we chose to model the interaction of TAC5-a with these two receptors. The chemical structure of TAC5-a was docked with the representative low-energy conformations of TLR7 and TLR8. Analysis of top-ranking docked poses revealed that the inhibitor stably occupies the cavity present at the lateral junction between two subunits (Figure 7A–D), predominantly forming hydrophobic contacts with the neighboring amino acids. The intermolecular interactions of TAC5-a largely resembled the agonistic ligand, R848, bound to the crystal structure of TLR7 (PDB ID: G5MH) [39]. The 2-aminopyridine ring of TAC5-a formed a π-interaction with F408, where the quinolone moiety of R848 interacts. The phenylamine group of TAC5-a was found entangled in a hydrophobic area defined by residues, F466, F351, and F449 of TLR7, where the ethoxymethyl group of R848 engages (Figure 7E). Similarly, the 2-aminopyridine ring of TAC5-a makes a π-stacking interaction with Y348 and its amino group forms a 2-Å hydrogen bond with the N262 of TLR8. A hydrophobic interaction was also observed between the phenylamine group of TAC5-a and TLR8 residues, F320, and F346 (Figure 7F). In the crystal structure of R848-bound TLR8 (PDB ID: 3W3L) [16], Y348 and F346 form the part of a characteristic hydrophobic cavity that is crucial for stabilizing the 2-ethoxymethyl substituent of R848 and is important for its agonistic activity. This indicates that the antagonistic activity of TAC5-a could mainly be attributed to its hydrophobic rings that stabilize the whole structure inside the polar-aromatic small molecule-binding cavity of TLR7/8.

## 4. Discussion

TLRs located on the endosomal membrane (TLR3, 7, 8, and 9) are crucial for protecting the host against various viral and bacterial infections. Notably, TLRs 7, 8, and 9 co-expression are essential for a sustained defense against pathogenic components or self-antigens released from damaged or stressed tissues/cells [46]. Malfunctions of these nucleic acid-sensing TLRs have been associated with several autoimmune pathologies, such as psoriasis and SLE [47]; however, the etiology of these diseases is still unclear [48,49]. Therefore, there is a growing need for the development of novel antagonists to reduce endosomal TLR-mediated disease progression. 

Here, we discovered a pyridine-containing chemical compound (TAC5) by utilizing QSAR models and obtained its equipotent 3-amino derivative (TAC5-a) via a synthetic route. Both compounds inhibited the secretion of proinflammatory cytokines triggered by TLR3/7/8/9 without affecting the cell surface TLRs (TLR1/2, TLR2/6, or TLR4). Based on sequence homology, TLRs 7/8/9 are grouped into one subfamily [50,51]; thus, achieving ligand selectivity against these structurally similar TLRs has been challenging [52]. This was evident in our study because of two particular reasons. First, in an attempt to find an agonist of human TLR8, we constructed a QSAR dataset containing several published TLR8 agonists. Nevertheless, the best hit exhibited inhibitory activity against all the endosomal TLRs in cell-based assays. Second, the initial lead (TAC5) suppressed CL075 and poly(dT) co-treatment-induced TLR8 signaling in mouse macrophages to some extent as compared to the CL075-induced case. poly(dT) is regarded as a specific activator of TLR8 when treated in combination with an imidazoquinoline, such as CL075 [44]. This indicates that mouse TLR8 could possibly be overstimulated by poly(dT), while TAC5 was able to block receptor activation. Intriguingly, a chemical database search revealed that TAC5, known as 2-amino-3-benzyloxypyridine (PubChem CID: 90334), was previously reported as a p38α MAPK inhibitor [53]. We also found that TAC5 significantly blocks the phosphorylation of ERK, JNK, and p-38 kinases (Figure 2B). The blockade of MAPKs, however, is a regular phenomenon frequently observed after TLR antagonist treatment. Since the cell-surface TLRs were unaffected in our experiments, we speculated that the inhibitors probably target the small molecule-binding cavities of the endosomal TLRs [16,39], thereby preventing receptor activation. Of all TAC5 analogs tested, TAC5-a showed a greater inhibitory effect as compared to TAC5-c or TAC5-e. We found that modifications at the R2 position of TAC5 (Figure 3) provided the ligand with improved bioactivity (TAC5-e); however, modifications at positions R1 (TAC5-c) or R3 (TAC5-e) resulted in the loss of activity. Structural superimposition of the lowest-energy docked conformations of TAC5-a and TAC5-c revealed that although the benzyl group (R1 position) of TAC5-c interacted with the small hydrophobic cavity lined by residues F351 and F466 in the TLR7 binding pocket (Supplementary Figure S4), it did not exhibit a noticeable inhibitory effect in vitro. This observation suggests that a pyridine ring (as in TAC5-a) seems more favorable for a stable receptor binding rather than a bulky phenylpyridine ring in the inhibitor. 

Recent medicinal chemistry efforts have resulted in the discovery of several antagonists [22,23,25,54] and agonists [55,56,57] of endosomal TLRs. Despite the breakthroughs in developing small molecular weight agonists/antagonists in vitro, a limited number of candidates have been evaluated in suitable disease models [33]. Dysregulation of TLR7/8/9, specifically TLR7 overexpression, has been closely associated with the pathology of psoriasis, a common inflammatory skin condition characterized by scaly reddish plaques caused by uncontrolled keratinocyte differentiation [58]. The TLR7/9 axis is also responsible for a systemic autoimmune disease, called SLE, which is characterized by painful and swollen joints, swollen lymph nodes, red rashes on the face, and fever [48]. Our in vivo studies showed that TAC5-a promisingly reduces the severity of psoriasis lesions in C57BL/6 mice induced by the application of IMQ, a TLR7 agonist. This IMQ-induced mouse model of psoriasis is being widely used to study the early and late phases of the disease [49,59], which imitates the human disorder well. In SLE, antibodies bound to self-DNA/RNAs from necrotic or apoptotic cells are recognized by immunoglobulin γ Fc region receptor IIa and internalized by plasmacytoid DCs as autoimmune complexes, which subsequently activate endosomal TLRs and upregulate IFN-α production [60,61]. Using lupus-prone MRL/lpr mice, we showed that TAC5-a markedly reduces disease markers, such as the level of circulating autoantibodies against dsDNA and ANAs in serum. Further, the albumin content in urine also decreased significantly after treatment with the inhibitor, demonstrating its ability to treat renal impairment associated with lupus nephritis [62]. The inhibition of IL-17 in the kidney and spleen was quite intriguing, as this cytokine is extensively expressed in the lungs, kidneys, liver, and spleen and has been associated with the activation and proliferation of B/T lymphocytes [63]. IL-17 family members are also considered important markers for diagnosis and pathogenesis of psoriasis, psoriatic arthritis, and SLE [64]. Although there were previously differing opinions about whether TLR8 is functional in mice [29,65], later research clarified that mouse TLR8 is indeed active and regulates the expression of TLR7 in DCs. TLR8^−/−^ deficient mice were hypersensitive to TLR7/8 agonist treatment (R848), showing several indicators of inflammation, such as splenomegaly, increased serum levels of autoantibodies against dsDNA or ribonucleoprotein, and GN [46]. Further, mouse TLR8-transfected HEK293 cells led to stronger and faster NF-κB-mediated secretion of proinflammatory cytokines in response to a combination of imidazoquinoline and poly-T oligonucleotides [44]. Researchers have now generated transgenic C57BL/6 mice expressing human TLR8 to study the role of this receptor in modulating the activities of concomitant TLRs, such as TLR7, and in regulating innate immunity as a whole [66,67]. 

There are numerous cases where TLR7/8, and occasionally TLR7/8/9, can bind to a common agonist or antagonist. For instance, Dynavax Technologies, Inc. designed oligonucleotide antagonists for TLR7/8/9 that are foreseen as treatments for autoimmune and inflammatory diseases [68]. In a novel approach, Kandimalla et al. [32] designed and synthesized oligonucleotide-based antagonists of TLR7/8/9 containing a 7-deaza-dG or arabino-G modification in the immune-stimulatory motif and 2’-O-methylribonucleotides in the immune-regulatory motif. The authors have evaluated the biological properties of these synthetic oligoribonucleotides against TLR7/8/9 in murine and human cell-based assays as well as in mice and non-human primate models [32]. Recently, Schmitt et al. [69] reported the identification of two trinucleotide motifs within a 9-mer oligoribonucleotide that are sufficient to antagonize TLR7 and TLR8 activation. IMO-8400, an antagonist of TLR7/8/9, has been found to reduce moderate-to-severe plaque psoriasis in clinical trial phase IIa patients [70]. Despite the progress in the deployment of oligonucleotide-based molecules, the small molecular weight, orally bioavailable poly-TLR inhibitors may have an advantage over oligonucleotide-based compounds. There is a possibility that oligonucleotides might interfere with distinct host pathways, deteriorating the host immune system [71]. Furthermore, the delivery of these large molecules to their desired target tissue site has been a matter of great concern [72]. Therefore, the development of small molecules for controlling autoimmune and inflammatory diseases is considered a safe and valuable alternative to oligonucleotide-based drugs [73]. Conclusively, the newly identified TAC5-a was found to be noncytotoxic and is a potential blocker of endosomal TLR-mediated downstream signal transduction with improved activity in animal models of psoriasis and SLE. This suggests that TAC5-a could possibly be developed as a low-cost alternative to its high molecular-weight oligonucleotide counterparts.

## Figures and Tables

**Table 1:** Summary of QSAR models considered in the current study for identifying the TLR3/7/8/9 antagonist.

Machine Learning Method	Descriptors	Prediction CCR	Accuracy	Sensitivity	Specificity
k-Nearest Neighbor	MOE 2D	0.711 ± 0.031	0.748	0.875	0.547
k-Nearest Neighbor	DragonX-H	0.733 ± 0.083	0.766	0.881	0.585
Random Forest	MOE 2D	0.737 ± 0.057	0.777	0.632	0.869
Random Forest	DragonX-H	0.717 ± 0.029	0.732	0.635	0.809
Support Vector Machine	MOE 2D	0.773 ± 0.031	0.762	0.721	0.794
Support Vector Machine	DragonX-H	0.705 ± 0.043	0.745	0.594	0.839

QSAR, quantitative structure–activity relationship; CCR, correct classification rate; TLR, Toll-like receptor; MOE, molecular operating environment.

## Supplementary Materials

The following are available online at https://www.mdpi.com/2073-4409/9/7/1648/s1, Figure S1: The overall workflow of the Quantitative Structure–Activity Relationship modeling, Figure S2: The chemical structures of TAC5 derivatives, Figure S3: The binding kinetics of TAC5 and inhibitory effects of TAC5-a and TAC5-c, Figure S4: Structural superimposition of TAC5-a and TAC5-c in the binding site of the TLR7 crystal structure. Table S1: List of chemical structures considered for QSAR model construction in the present study, Table S2: List of 13 small molecular weight compounds selected for experimental validation, and Chemical synthesis and characterization of TAC5 derivatives.
