# Supplementary material for: A Novel Small-Molecule Inhibitor of Endosomal TLRs Reduces Inflammation and Alleviates Autoimmune Disease Symptoms in Murine Models
Source: Cells. 2020 Jul 9;9(7):1648. doi: 10.3390/cells9071648 (PMC7407930; doi:10.3390/cells9071648)
Supplement: Supplementary file 1 [file cells-09-01648-s001.pdf]

Supplementary Materials

A novel small-molecule inhibitor of endosomal TLRs reduces inflammation and alleviates autoimmune disease symptoms in murine models

Mahesh Chandra Patra[1†], Asma Achek[1†], Gi-Young Kim[1†], Suresh Panneerselvam[1], Hyeon-Jun Shin[1], Wook-Yong Baek[2], Wang Hee Lee[1], June Sung[1], Uisuk Jeong[1], Eun-Young Cho[1], Wook Kim[1], Eunha Kim[1], Chang-Hee Suh[2] and Sangdun Choi[1]*

[1]Department of Molecular Science and Technology, Ajou University, Suwon, 16499, Korea

[2]Department of Rheumatology, Ajou University School of Medicine, Suwon, 16499, Korea

*Correspondence: sangdunchoi@ajou.ac.kr

†These authors have contributed equally to this work.

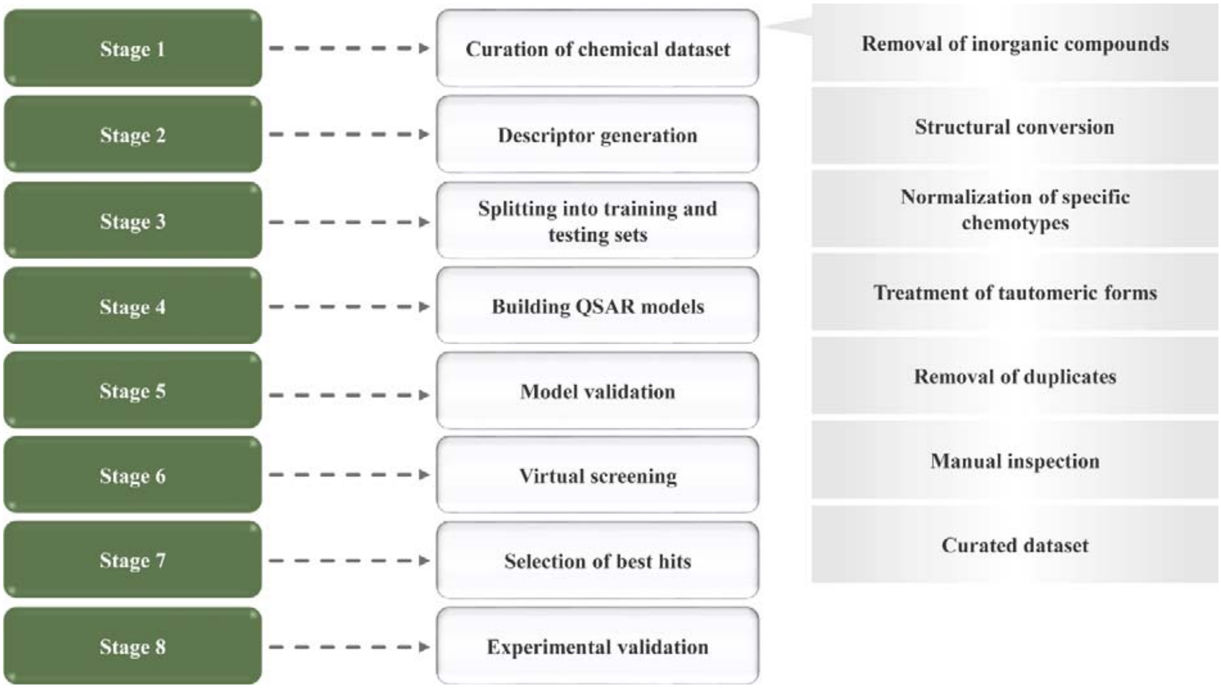

Figure S1. The overall workflow of the Quantitative Structure-Activity Relationship modeling. The curation of a chemical dataset (Stage 1) was further subdivided into several independent stages.

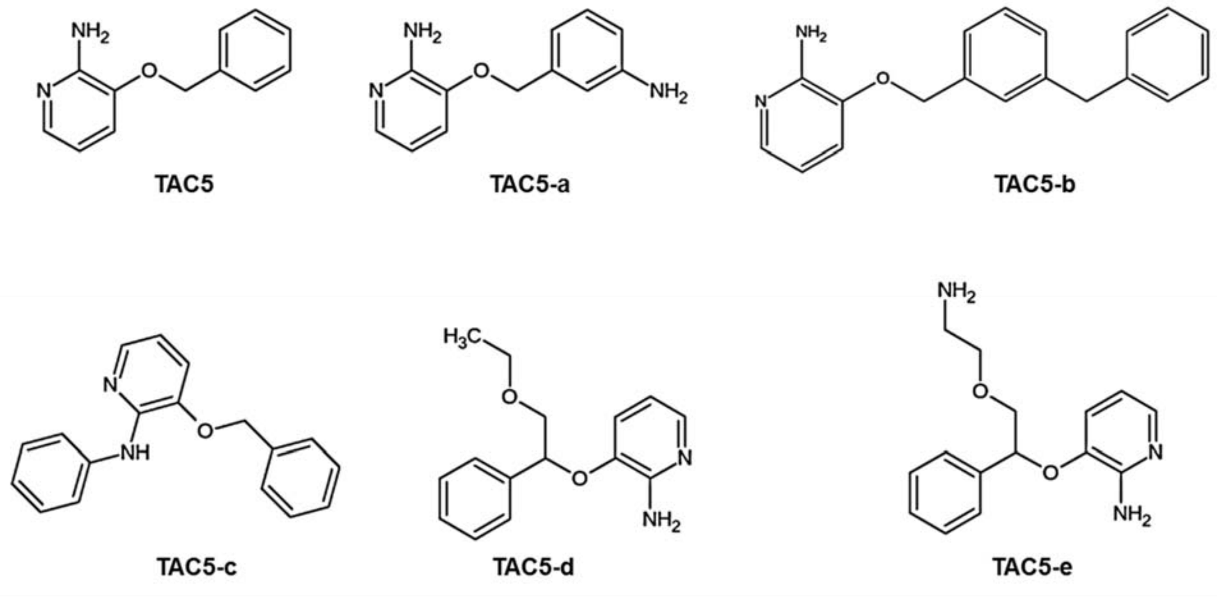

Figure S2. The chemical structures of TAC5 derivatives.

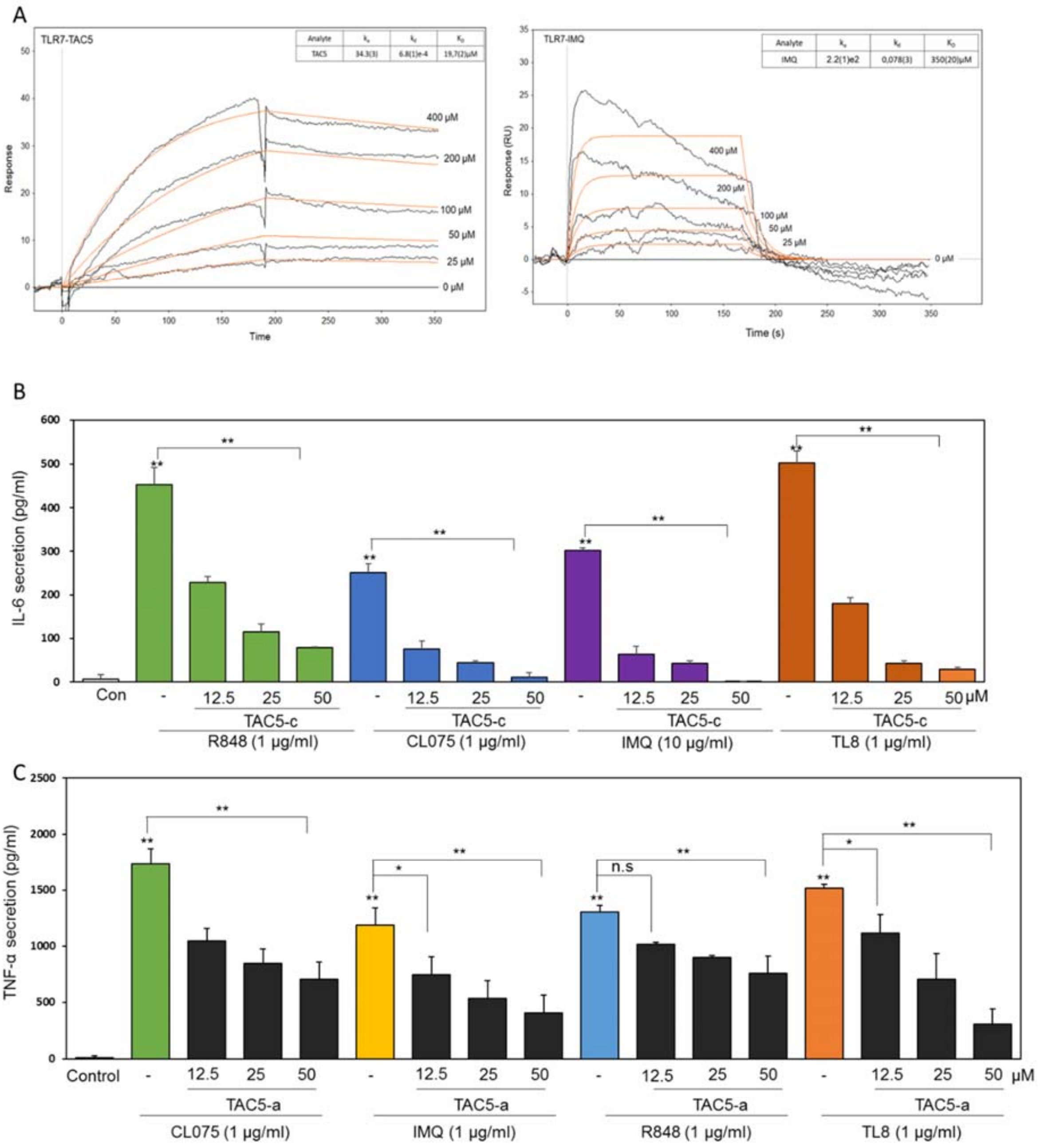

Figure S3. The binding kinetics of TAC5 and inhibitory effects of TAC5-a and TAC5-c. (**A**) SPR sensorgram illustrating the binding of TAC5 and IMQ to TLR7. (**B**) The inhibitory effects of TAC5-c on IL-6 secretion in RAW264.7 cells. (**C**) The inhibitory effects of TAC5-a on TNF-α secretion. The evaluation of the TAC5-a effect on the secretion of TNF-α from RAW264.7 cells after stimulating TLR7/8 with R848 or CL075, TLR7 with imiquimod, and TLR8 with TL8-506 ligands.

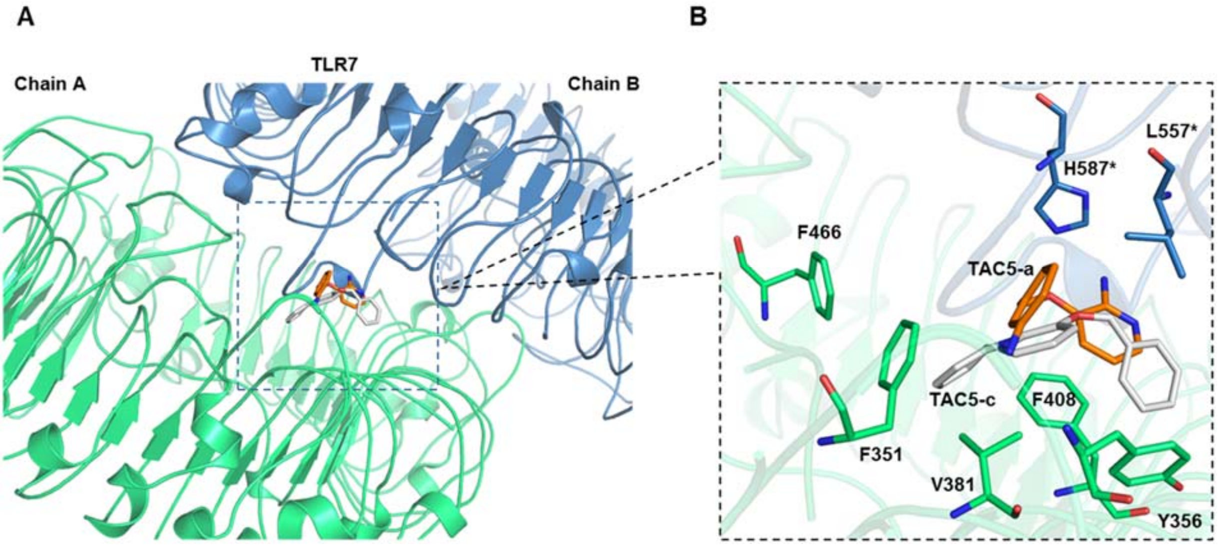

Figure S4. Structural superimposition of TAC5-a and TAC5-c in the binding site of the TLR7 crystal structure. (**A**) Overview of representative superimposed ligands in the ligand-binding site of the TLR7 extracellular domain. (**B**) A magnified view of the detailed intermolecular interaction of both TAC5-a and TAC5-c with the residues of TLR7.

Table S1. List of chemical structures considered for QSAR model construction in the present study.

#	SMILES Notation	Name	Status	Reference	Concentration
1	o1c2c(c(Nc3ncccc3)c1NC1CCCCC1)c(c[nH+]c2C)CO	26_2012	Active	[58]	6.93 µM
2	o1c2c(c(Nc3nccnc3)c1NC1CCCCC1)c(c[nH+]c2C)CO	27_2012	Active	[58]	10.58 µM
3	o1c2c(c(Nc3ncccc3)c1Nc1ccccc1)c(c[nH+]c2C)CO	28_2012	Active	[58]	4.91 µM
4	o1c2c(c(Nc3nccnc3)c1Nc1ccccc1)c(c[nH+]c2C)CO	29_2012	Active	[58]	9.79 µM
5	o1c2c(c(Nc3ccccc3)c1Nc1ccccc1)c(cnc2C)CO	36_2012	Active	[58]	1.68 µM
6	o1c2c(c(Nc3ncccc3)c1NCCCC)c(c[nH+]c2C)CO	37a_2012	Active	[58]	9.38 µM
7	o1c2c(c(Nc3ncccc3)c1NCCCCC)c(c[nH+]c2C)CO	37b_2012	Active	[58]	5.81 µM
8	o1c2c(c(Nc3ncccc3)c1NC(CCC)C)c(c[nH+]c2C)CO	37d_2012	Active	[58]	9.01 µM
9	[Si](Nc1oc2c(c1Nc1ncccc1)c(c[nH+]c2C)CO)(C)(C)C	37f_2012	Active	[58]	4.99 µM
10	o1c2c(c(Nc3ncccc3)c1NCCC(OC(C)(C)C)=O)c(c[nH+]c2C)CO	37i_2012	Active	[58]	7.64 µM
11	o1c2c(c(Nc3ncccc3)c1NC(C)c1ccccc1)c(c[nH+]c2C)CO	37m_2012	Active	[58]	4.27 µM
12	o1c2c(c(Nc3ccccc3)c1NCCCCC)c(c[nH+]c2C)CO	38a_2012	Active	[58]	2.25 µM
13	Fc1cc(Nc2c3c(oc2NCCCCC)c([nH+]cc3CO)C)ccc1	38b_2012	Active	[58]	0.37 µM
14	o1c2c(c(Nc3cc([N+](=O)[O-])ccc3)c1NCCCCC)c(c[nH+]c2C)CO	38c_2012	Active	[58]	0.85 µM
15	[sH+]1c2c(nc1C)c(nc1c2cccc1)N	8a_2013	Active	[76]	2.06 µM
16	[sH+]1c2c(nc1CC)c(nc1c2cccc1)N	8b_2013	Active	[76]	0.81 µM
17	[sH+]1c2c(nc1CCC)c(nc1c2cccc1)N	8c_2013	Active	[76]	1.32 µM
18	[sH+]1c2c(nc1CCCC)c(nc1c2cccc1)N	8d_2013	Active	[76]	0.41 µM
19	[sH+]1c2c(nc1CCCCC)c(nc1c2cccc1)N	8e_2013	Active	[76]	1.94 µM
20	[sH+]1c2c(nc1C(C)C)c(nc1c2cccc1)N	8i_2013	Active	[76]	10.85 µM
21	[sH+]1c2c(nc1CC(C)C)c(nc1c2cccc1)N	8j_2013	Active	[76]	1.55 µM
22	[sH+]1c2c(nc1C(CC)C)c(nc1c2cccc1)N	8n_2013	Active	[76]	2.77 µM
23	[sH+]1c2c(nc1CC(CC)C)c(nc1c2cccc1)N	8o_2013	Active	[76]	2.51 µM
24	[sH+]1c2c(nc1CCC(F)(F)F)c(nc1c2cccc1)N	8p_2013	Active	[76]	1.61 µM

25	[sH+]1c2c(nc1CCCC(F)(F)F)c(nc1c2cccc1)N	8q_2013	Active	[76]	6.29 µM
26	[sH+]1c2c(nc1CCC)c(nc1cc(N)ccc12)N	10_2013	Active	[76]	3.88 µM
27	[sH+]1c2c(nc1CCC)c(nc1cc([N-][N+]#N)ccc12)N	11_2013	Active	[76]	2.06 µM
28	[sH+]1c2c(nc1CCC)c(nc1c2cc(-n2nnc([Si](C)(C)C)c2)cc1)N	12f_2013	Active	[76]	9.84 µM
29	[sH+]1c2c(nc1CCC)c(nc1c2cccc1)NC(=O)C	15e_2013	Active	[76]	0.88 µM
30	[sH+]1c2c(nc1CCC)c(nc1c2cccc1)NC(=O)CCC	15f_2013	Active	[76]	1.14 µM
31	[sH+]1c2c(nc1CCC)c(nc1c2cccc1)NC(=O)C[N-][N+]#N	15j_2013	Active	[76]	1.2 µM
32	[sH+]1c2c(nc1CCC)c(nc1c2cccc1)NC(=O)CC[N-][N+]#N	15k_2013	Active	[76]	2.23 µM
33	[sH+]1c2c(nc1CCC)c(nc1c2cccc1)NC(=O)CCC#C	15l_2013	Active	[76]	1.2 µM
34	[sH+]1c2c(nc1CCC)c(nc1c2cccc1)NS(=O)(=O)C	15r_2013	Active	[76]	10.6 µM
35	[sH+]1c2c(nc1CCCC)c(nc1c2cccc1)NC=O	18a_2013	Active	[76]	2.31 µM
36	[sH+]1c2c(nc1CCCC)c(nc1c2cccc1)NC(=O)CCC	18c_2013	Active	[76]	0.42 µM
37	O(Cc1nc2c(n1CC(O)(C)C)c1c(nc2N)cccc1)CC	R848_2015	Active	[77]	66.6 ng/ml
38	OC(Cn1c2c(nc1CCCC)c(nc1c2cccc1)N)(C)C	Hybrid-2_2015	Active	[77]	2.5 ng/ml
39	n1c2c(n(Cc3ccc(cc3)CN)c1CCCC)c1c(nc2N)cccc1	para-amine_2015	Active	[77]	4.02 ng/ml
40	[NH3+]Cc1cc(ccc1)Cn1c2c(nc1CCCC)c(nc1c2cccc1)N	Meta-amine_2015	Active	[77]	29.4 ng/ml
41	n1c(cn(CCCCC)c1N)-c1ccccc1	17a_2016	Active	[78]	2.48 µM
42	n1c(cn(CCCCC)c1N)-c1ccccc1C	17b_2016	Active	[78]	2.5 µM
43	n1c(cn(CCCCC)c1N)-c1ccccc1CC	17c_2016	Active	[78]	2.5 µM
44	n1c(cn(CCCCC)c1N)-c1cc(ccc1)C	17d_2016	Active	[78]	2 µM
45	n1c(cn(CCCCC)c1N)-c1ccc(cc1)C	17e_2016	Active	[78]	2.5 µM
46	n1c(cn(CCCCC)c1N)-c1cccc(C)c1C	17f_2016	Active	[78]	1.5 µM
47	n1c(cn(CCCCC)c1N)-c1ccc(cc1C)C	17g_16	Active	[78]	1.5 µM
48	n1c(cn(CCCCC)c1N)-c1cc(ccc1C)C	17h_2016	Active	[78]	1.5 µM
49	n1c(cn(CCCCC)c1N)-c1c(cccc1C)C	17i_2016	Active	[78]	2.5 µM
50	n1c(cn(CCCCC)c1N)Cc1ccccc1	18a_1_2016	Active	[78]	2.7 µM
51	n1c(cn(CCCCC)c1N)CCc1ccccc1	18b_1_2016	Active	[78]	1.8 µM

52	n1c(cn(CCCCC)c1N)-c1c2c(ccc1)cccc2	18c_1_2016	Active	[78]	1.5 µM
53	OCc1ccccc1-c1nc(n(c1)CCCCC)N	19d_2016	Active	[78]	8.05 µM
54	n1c(cn(CCCCC)c1N)-c1ccc(N)cc1	19e_2016	Active	[78]	8 µM
55	O(C(=O)c1ccc(cc1)-c1nc(n(c1)CCCCC)N)C	19g_2016	Active	[78]	4.04 µM
56	O(C)c1ccccc1-c1nc(n(c1)CCCCC)N	20a_2016	Active	[78]	1.6 µM
57	O(C)c1cc(ccc1)-c1nc(n(c1)CCCCC)N	20b_2016	Active	[78]	2.2 µM
58	O(C)c1ccc(cc1)-c1nc(n(c1)CCCCC)N	20c_2016	Active	[78]	2.94 µM
59	O(C)c1cc(C)c(cc1)-c1nc(n(c1)CCCCC)N	20d_2016	Active	[78]	1.5 µM
60	O(C)c1c(cccc1C)-c1nc(n(c1)CCCCC)N	20e_2016	Active	[78]	1.5 µM
61	O(C)c1c(cccc1OC)-c1nc(n(c1)CCCCC)N	20f_2016	Active	[78]	1.5 µM
62	O(C)c1c(OC)cc(cc1OC)-c1nc(n(c1)CCCCC)N	20g_2016	Active	[78]	3.08 µM
63	Clc1ccccc1-c1nc(n(c1)CCCCC)N	21a_2016	Active	[78]	1.36 µM
64	Clc1cc(ccc1)-c1nc(n(c1)CCCCC)N	21b_2016	Active	[78]	1.93 µM
65	Clc1ccc(cc1)-c1nc(n(c1)CCCCC)N	21c_2016	Active	[78]	6.83 µM
66	Fc1ccccc1-c1nc(n(c1)CCCCC)N	21d_2016	Active	[78]	2.35 µM
67	Fc1cc(ccc1)-c1nc(n(c1)CCCCC)N	21e_2016	Active	[78]	2.57 µM
68	Fc1ccc(cc1)-c1nc(n(c1)CCCCC)N	21f_2016	Active	[78]	1.96 µM
69	Fc1cc(C)c(cc1)-c1nc(n(c1)CCCCC)N	21g_2016	Active	[78]	2.01 µM
70	FC(F)(F)c1ccccc1-c1nc(n(c1)CCCCC)N	21h_2016	Active	[78]	1.64 µM
71	FC(F)(F)c1cc(ccc1)-c1nc(n(c1)CCCCC)N	21i_2016	Active	[78]	1.98 µM
72	FC(F)(F)c1ccc(cc1)-c1nc(n(c1)CCCCC)N	21j_2016	Active	[78]	2.48 µM
73	Ic1c(nc(nc1NCCCC)N)C	4b_2016_2	Active	[79]	1.64 µM
74	Ic1c(nc(nc1NCCCCC)N)C	4c_2016_2	Active	[79]	3.7 µM
75	Brc1c(nc(nc1NCCCC)N)C	6b	Active	[79]	8.5 µM
76	n1c(C)c(CCCN)c(nc1N)NCCCC	27a	Active	[79]	2.31 µM
77	n1c(C)c(CCCCN)c(nc1N)NCCCC	27b	Active	[79]	0.3 µM
78	n1c(C)c(CCCCCN)c(nc1N)NCCCC	27c	Active	[79]	0.3 µM

79	o1c2c(c(Nc3[nH+]cccc3)c1Nc1ccccc1)c(c[nH+]c2C)CO	28	Active	[79]	6.7 µM
80	n1c(-c2ccccc2)c(CCCCN)c(nc1N)NCCCC	32b	Active	[79]	2.7 µM
81	n1c(-c2ccccc2)c(CCCCCN)c(nc1N)NCCCC	32c	Active	[79]	2.35 µM
82	n1c2c(cccc2)c(Cc2cc(ccc2)CN)c(CCCCC)c1N	9a_2015	Active	[80]	150 nM
83	[NH3+]Cc1ccc(cc1)Cc1c2c(nc(N)c1CCCCC)cccc2	9b_2015	Active	[80]	120 nM
84	[NH3+]CCCCc1c2c(nc(N)c1CCCCC)cccc2	14a_2015	Active	[80]	190 nM
85	n1c2c(cccc2)c(CCCCCN)c(CCCCC)c1N	14b_2015	Active	[80]	250 nM
86	n1c2c(cc(CCCCC)c1N)c(ccc2)Cc1cc(ccc1)CN	18a_2015	Active	[80]	49 nM
7	n1c2c(cc(CCCCC)c1N)c(ccc2)Cc1ccc(cc1)CN	18b_2015	Active	[80]	38 nM
88	n1c2c(cc(CCCCC)c1N)c(ccc2)Cc1ccccc1CN	18c_2015	Active	[80]	1000 nM
89	[NH3+]Cc1ccc(cc1)-c1c2cc(CCCCC)c(nc2ccc1)N	20b_2015	Active	[80]	699 nM
90	n1c2c(cc(CCCCC)c1N)c(ccc2)CCCN	23_b_2015	Active	[80]	91 nM
91	n1c2c(cc(CCCCC)c1N)c(ccc2)CCCCN	34a_2_2015	Active	[80]	27 nM
92	n1c2c(cc(CCCCC)c1N)c(ccc2)CCCCCN	34b_2_2015	Active	[80]	9 nM
93	n1c2c(cc(CCCCC)c1N)c(ccc2)CCCCCCN	34c_2_2015	Active	[80]	56 nM
94	O=C(N)CCCc1c2cc(CCCCC)c(nc2ccc1)N	34d_2_2015	Active	[80]	2181 nM
95	n1c2c(cc(CCCCC)c1N)c(ccc2)CCCCNC(N)=N	34e_2_2015	Active	[80]	2862 nM
96	n1c2c(cc(CCCCC)c1N)cc(cc2)CCCCN	35a_2_2015	Active	[80]	727 nM
97	n1c2c(cc(CCCCC)c1N)cc(cc2)CCCCCN	35b_2_2015	Active	[80]	519 nM
98	n1c2c(cc(CCCCC)c1N)cc(cc2)CCCCCCN	35c_2_2015	Active	[80]	1016 nM
99	n1c2cc(ccc2cc(CCCCC)c1N)CCCCN	36a_2_2015	Active	[80]	60 nM
100	n1c2cc(ccc2cc(CCCCC)c1N)CCCCCN	36b_2_2015	Active	[80]	50 nM
101	n1c2cc(ccc2cc(CCCCC)c1N)CCCCCCN	36c_2_2015	Active	[80]	85 nM
102	n1c2c(cc(CCCCC)c1N)c(cc(c2)CCCCCN)CCCCCN	43_2015	Active	[80]	621 nM
103	n1c2c(n(CCCC)c1N)cccc2	8a_3	Active	[81]	7.3 µM
104	n1c2c(n(CCCCC)c1N)cccc2	8b_3	Active	[81]	3.23 µM
105	n1c2c(n(CCCCCC)c1N)cccc2	8c_3	Active	[81]	3.95 µM

106	n1c2c3c(ccc2n(CCCCC)c1N)cccc3	23_3_3	Active	[81]	3.16 µM
107	n1c2c(n(CCCCCC)c1N)cccc2C	31a_3	Active	[81]	1.13 µM
108	n1c2cc(ccc2n(CCCCCC)c1N)C	31b_3	Active	[81]	4.57 µM
109	n1c2c(n(CCCCCC)c1N)cc(cc2)C	31c_3	Active	[81]	7.21 µM
110	n1c2c(n(CCCCCC)c1N)c(ccc2)C	31d_3	Active	[81]	6.61 µM
111	O(C)c1c2nc(n(c2ccc1)CCCCCC)N	31e_3	Active	[81]	3.74 µM
112	n1c2c(n(CCCCCC)c1N)cccc2CC	31j_3	Active	[81]	1.65 µM
113	n1c2c(n(CCCCCC)c1N)cccc2N(C)C	31k_3	Active	[81]	7.12 µM
114	Oc1c2nc(n(c2ccc1)CCCCCC)N	36_3	Active	[81]	5.01 µM
115	n1c2c(n(CCCCCC)c1N)cccc2N	40_3	Active	[81]	6.6 µM
116	O(CCCC)c1cc2c(nc1N)cccc2	6_4	Active	[82]	2.18 µM
117	n1c2c(cc(NCCCC)c1N)cccc2	9_4	Active	[82]	4.28 µM
118	S(CCCC)c1cc2c(nc1N)cccc2	12_4	Active	[82]	4.16 µM
119	n1c2c(cc(CCCC)c1N)cccc2	14a_4	Active	[82]	0.41 µM
120	n1c2c(cc(CCCCC)c1N)cccc2	14b_4	Active	[82]	0.2 µM
121	n1c2c(cc(C=CCCC)c1N)cccc2	14d_4	Active	[82]	2.67 µM
122	n1c2c(cc(CCCC=C)c1N)cccc2	14e_4	Active	[82]	0.49 µM
123	O(CCC)c1cc2c(nc1N)cccc2	6c_4	Active	[82]	5 µM
124	O(CCCCC)c1cc2c(nc1N)cccc2	6d_4	Active	[82]	7 µM
125	O(CC(CC)C)c1cc2c(nc1N)cccc2	6i_4	Active	[82]	10 µM
126	OCCCn1c2c(nc1CCCCCCCCC)c1c(nc2)cccc1	6d_2011	Active	[56]	4.81 µM
127	n1c2c3c(ncc2n(CC#C)c1CCCCCCCCC)cccc3	7d_2011	Active	[56]	10.07 µM
128	O(C)c1ccc(cc1)Cn1c2c(nc1-c1ccccc1)c1c([n+](c2)Cc2ccc(OC)cc2)cccc1	12_2011	Active	[56]	4.55 µM
129	o1c2c(c(Nc3ncccc3)c1NC(C)(C)C)c(c[nH+]c2C)CO	24_2012	Inactive	[58]	N/A
130	o1c2c(c(Nc3nccnc3)c1NC(C)(C)C)c(c[nH+]c2C)CO	25_2012	Inactive	[58]	N/A
131	O(c1cncc(O)c1-c1[nH]c2[n+](cccc2)c1NC1CCCCC1)c1ccccc1	35_2012	Inactive	[58]	N/A
132	o1c2c(c(Nc3ncccc3)c1NC(C)C)c(c[nH+]c2C)CO	37c_2012	Inactive	[58]	N/A

133	o1c2c(c(Nc3nccnc3)c1NC(CC(C)(C)C)(C)C)c(c[nH+]c2C)CO	37e_2012	Inactive	[58]	N/A
134	o1c2c(c(Nc3ncccc3)c1NCCN1CCOCC1)c(c[nH+]c2C)CO	37g_2012	Inactive	[58]	N/A
135	o1c2c(c(Nc3ncccc3)c1NCC(OCC)=O)c(c[nH+]c2C)CO	37h_2012	Inactive	[58]	N/A
136	P(OCC)(OCC)([O-])=[CH]Nc1oc2c(c1Nc1ncccc1)c(c[nH+]c2C)CO	37j_2012	Inactive	[58]	N/A
137	o1c2c(c(Nc3ncccc3)c1Nc1ccc(OC)cc1)c(c[nH+]c2C)CO	37k_2012	Inactive	[58]	N/A
138	Clc1cccc(C)c1Nc1oc2c(c1Nc1ncccc1)c(c[nH+]c2C)CO	37l_2012	Inactive	[58]	N/A
139	o1c2c(c(Nc3ncccc3)c1N)c(c[nH+]c2C)CO	37n_2012	Inactive	[58]	N/A
140	[sH+]1c2c(nc1CCCCCC)c(nc1c2cccc1)N	8f_2013	Inactive	[76]	N/A
141	[sH+]1c2c(nc1CCCCCCC)c(nc1c2cccc1)N	8g_2013	Inactive	[76]	N/A
142	[sH+]1c2c(nc1CCCCCCCCC)c(nc1c2cccc1)N	8h_2013	Inactive	[76]	N/A
143	[sH+]1c2c(nc1CC(C)(C)C)c(nc1c2cccc1)N	8k_2013	Inactive	[76]	N/A
144	[sH+]1c2c(nc1CCC(C)C)c(nc1c2cccc1)N	8l_2013	Inactive	[76]	N/A
145	[sH+]1c2c(nc1CCC(C)(C)C)c(nc1c2cccc1)N	8m_2013	Inactive	[76]	N/A
146	[sH+]1c2c(nc1CCC)c(nc1cc([N+](=O)[O-])ccc12)N	9_2013	Inactive	[76]	N/A
147	[sH+]1c2c(nc1CCC)c(nc1c2cc(-n2nnc(c2)C(O)(C)C)cc1)N	12a_2013	Inactive	[76]	N/A
148	[sH+]1c2c(nc1CCC)c(nc1c2cc(-n2nnc(c2)CO)cc1)N	12b_2013	Inactive	[76]	N/A
149	[sH+]1c2c(nc1CCC)c(nc1c2cc(-n2nnc(c2)-c2ccccc2)cc1)N	12c_2013	Inactive	[76]	N/A
150	[sH+]1c2c(nc1CCC)c(nc1c2cc(-n2nnc(c2)CCC)cc1)N	12d_2013	Inactive	[76]	N/A
151	[sH+]1c2c(nc1CCC)c(nc1c2cc(-n2nnc(c2)CCCCCC)cc1)N	12e_2013	Inactive	[76]	N/A
152	[sH+]1c2c(nc1CCC)c(nc1c2cc(-n2nncc2)cc1)N	12g_2013	Inactive	[76]	N/A
153	[sH+]1c2c(nc1CCC)c(nc1c2cc(cc1)CCCCCC)N	13a_2013	Inactive	[76]	N/A
154	[sH+]1c2c(nc1CCC)c(nc1c2cc(cc1)C(=O)CCC)N	13b_2013	Inactive	[76]	N/A
155	Brc1cc2c(nc(N)c3nc([sH+]c23)CCC)cc1	14_2013	Inactive	[76]	N/A
156	[sH+]1c2c(nc1CCC)c(nc1c2cccc1)NCCCC	15a_2013	Inactive	[76]	N/A
157	[sH+]1c2c(nc1CCC)c(nc1c2cccc1)NCCCCCC	15b_2013	Inactive	[76]	N/A
158	[sH+]1c2c(nc1CCC)c(nc1c2cccc1)NCCCCCCCCCCCCCCCC	15c_2013	Inactive	[76]	N/A
159	[sH+]1c2c(nc1CCC)c(nc1c2cccc1)NC=O	15d_2013	Inactive	[76]	N/A

160	[sH+]1c2c(nc1CCC)c(nc1c2cccc1)NC(=O)CCCCCCC	15g_2013	Inactive	[76]	N/A
161	[sH+]1c2c(nc1CCC)c(nc1c2cccc1)NC(=O)CCCCCCCCCCCCCCC	15h_2013	Inactive	[76]	N/A
162	[sH+]1c2c(nc1CCC)c(nc1c2cccc1)NC(=O)CCCCC	15i_2013	Inactive	[76]	N/A
163	[sH+]1c2c(nc1CCC)c(nc1c2cccc1)NC(OCC)=O	15n_2013	Inactive	[76]	N/A
164	[sH+]1c2c(nc1CCC)c(nc1c2cccc1)NC(OCCCC)=O	15o_2013	Inactive	[76]	N/A
165	[sH+]1c2c(nc1CCC)c(nc1c2cccc1)NC(OCCCCCCCC)=O	15p_2013	Inactive	[76]	N/A
166	[sH+]1c2c(nc1CCC)c(nc1c2cccc1)NC(=O)N	15q_2013	Inactive	[76]	N/A
167	[sH+]1c2c(nc1CCC)c(nc1c2cccc1)NS(=O)(=O)CC	15s_2013	Inactive	[76]	N/A
168	[sH+]1c2c(nc1CCC)c(nc1c2cccc1)NS(=O)(=O)CCC	15t_2013	Inactive	[76]	N/A
169	[sH+]1c2c(nc1CCC)c(nc1c2cccc1)NS(=O)(=O)CCCC	15u_2013	Inactive	[76]	N/A
170	[sH+]1c2c(nc1CCC)c(nc1c2cccc1)NS(=O)(=O)c1ccc(cc1)C	15v_2013	Inactive	[76]	N/A
171	[sH+]1c2c(nc1CCC)c(nc1c2cccc1)NP(OCC)(OCC)=O	15w_2013	Inactive	[76]	N/A
172	[sH+]1c2c(nc1CCC)c(nc1c2cccc1)NC(N)=N	17_2013	Inactive	[76]	N/A
173	[sH+]1c2c(nc1CCCC)c(nc1c2cccc1)NC(=O)C	18b_2013	Inactive	[76]	N/A
174	n1cc(n(CCCCC)c1N)-c1ccccc1	7_2016	Inactive	[78]	N/A
175	O(Cc1ccccc1)c1ccccc1-c1nc(n(c1)CCCCC)N	18f_2016	Inactive	[78]	N/A
176	Ic1c(nc(nc1NCCC)N)C	4a_2016_2	Inactive	[79]	N/A
177	Ic1c(nc(nc1NCCCCCC)N)C	4d	Inactive	[79]	N/A
178	Ic1c(nc(nc1N(CCCC)C)N)C	4e	Inactive	[79]	N/A
179	Ic1c(nc(nc1N(Cc1ccccc1)CCCC)N)C	4f	Inactive	[79]	N/A
180	Ic1c(nc(nc1OCCCC)N)C	4g	Inactive	[79]	N/A
181	Ic1c(nc(nc1SCCCC)N)C	4h	Inactive	[79]	N/A
182	n1c(cc(nc1N)C)C#CCCC	4i	Inactive	[79]	N/A
183	n1c(cc(nc1N)C)CCCCC	4j	Inactive	[79]	N/A
184	Ic1c(nc(nc1C)N)CCCCC	4k	Inactive	[79]	N/A
185	Ic1cnc(nc1NCCCC)N	17	Inactive	[79]	N/A
186	n1c(NCCCC)cc(nc1N)N	18	Inactive	[79]	N/A

187	O(C)c1nc(nc(NCCCC)c1)N	19	Inactive	[79]	N/A
188	Clc1nc(nc(NCCCC)c1)N	20	Inactive	[79]	N/A
189	Ic1c(nc(nc1N)N)NCCCC	21	Inactive	[79]	N/A
190	Ic1c(nc(nc1OC)N)NCCCC	22	Inactive	[79]	N/A
191	Ic1c(nc(nc1Cl)N)NCCCC	23	Inactive	[79]	N/A
192	o1c2c(c(Nc3[nH+]ccnc3)c1Nc1ccccc1)c(c[nH+]c2C)CO	29	Inactive	[79]	N/A
193	n1c(-c2ccccc2)c(CCCN)c(nc1N)NCCCC	32a	Inactive	[79]	N/A
194	n1c2c(cc(CCCCC)c1N)c(ccc2)Cc1cc(ccc1)C#N	17a_1_2015	Inactive	[80]	N/A
195	n1c2c(cc(CCCCC)c1N)c(ccc2)Cc1ccc(cc1)C#N	17b_2015	Inactive	[80]	N/A
196	n1c2c(cc(CCCCC)c1N)c(ccc2)Cc1ccccc1	17d_2015	Inactive	[80]	N/A
197	O=C(N)c1cc(ccc1)Cc1c2cc(CCCCC)c(nc2ccc1)N	18d_2015	Inactive	[80]	N/A
198	n1c2c(cc(CCCCC)c1N)c(ccc2)-c1cc(ccc1)CN	20a_2015	Inactive	[80]	N/A
199	n1c2c(cc(CCCCC)c1N)cccc2CCCCN	37_2015	Inactive	[80]	N/A
200	OC1(c2c(N=C1N)cccc2)CCCC	5_3	Inactive	[80]	N/A
201	n1c2c(n(Cc3ccccc3)c1N)cccc2	8d_3	Inactive	[80]	N/A
202	n1c2c(n(Cc3cc(N)ccc3)c1N)cccc2	8f_3	Inactive	[80]	N/A
203	O(CCCC)C(=O)n1c2c(nc1N)cccc2	9_3	Inactive	[80]	N/A
204	O=C(Nc1nc2c(n1CCCCCC)cccc2)C	10_3	Inactive	[80]	N/A
205	n1c2c(n(CCCCC)c1N)cc1c(c2)cccc1	13_3	Inactive	[80]	N/A
206	n1c2c(n(CCCCC)c1N)c1c(cc2)cccc1	18_3	Inactive	[80]	N/A
207	n1c2ncccc2n(CCCCCC)c1N	27a_3	Inactive	[80]	N/A
208	n1c2c(n(CCCCCC)c1N)ccnc2	27b_3	Inactive	[80]	N/A
209	n1c2c(n(CCCCCC)c1N)cncc2	27c_3	Inactive	[80]	N/A
210	n1c2cccnc2n(CCCCCC)c1N	27d_3	Inactive	[80]	N/A
211	Fc1c2nc(n(c2ccc1)CCCCCC)N	31f_3	Inactive	[80]	N/A
212	Clc1c2nc(n(c2ccc1)CCCCCC)N	31g_3	Inactive	[80]	N/A
213	FC(F)(F)c1c2nc(n(c2ccc1)CCCCCC)N	31h_3	Inactive	[80]	N/A

214	Brc1c2nc(n(c2ccc1)CCCCCC)N	31i_3	Inactive	[80]	N/A
215	n1c2c(n(CCCCCC)c1N)cccc2-c1ccccc1	31l_3	Inactive	[80]	N/A
216	n1c2c(n(CCCCCC)c1N)cccc2Cc1ccccc1	31m_3	Inactive	[80]	N/A
217	O(Cc1ccccc1)c1c2nc(n(c2ccc1)CCCCCC)N	35_3	Inactive	[80]	N/A
218	O=[N+]([O-])c1c2nc(n(c2ccc1)CCCCCC)N	39_3	Inactive	[80]	N/A
219	n1c2c(cc(CCCCC)c1N)cccc2	14c_4	Inactive	[82]	N/A
220	n1c2c(cc(CCCCC)c1N)cccc2	21a_4	Inactive	[82]	N/A
221	n1c2c(cccc2)c(CC)c(CCCCC)c1N	21b_4	Inactive	[82]	N/A
222	n1c2c(cccc2)c(CCC(C)C)c(CCCCC)c1N	21c_4	Inactive	[82]	N/A
223	O(CCCC)c1cc(nc2c1cccc2)N	24a_4	Inactive	[82]	N/A
224	O(CCCCC)c1cc(nc2c1cccc2)N	24b_4	Inactive	[82]	N/A
225	n1c2c(cccc2)c(cc1N)CCCCC	27a_4_4	Inactive	[82]	N/A
226	n1c2c(cccc2)c(cc1N)CCCC	27a_4	Inactive	[82]	N/A
227	[n+]12c([nH]c(-c3ccccc3)c1NC1CCCCC1)c(ncc2)N	5a_5	Inactive	[58]	N/A
228	[n+]12c([nH]c(-c3ccc(cc3)-c3ccccc3)c1NC1CCCCC1)c(ncc2)N	5b_5	Inactive	[58]	N/A
229	[n+]12c([nH]c(CCCC)c1Nc1ccccc1)c(ncc2)N	5c_5	Inactive	[58]	N/A
230	O(C)c1ccc(Nc2[n+]3c([nH]c2CCCC)c(ncc3)N)cc1	5d_5	Inactive	[58]	N/A
231	[n+]12c([nH]c(-c3ccccc3)c1NC(C)(C)C)c(ncc2)N	6	Inactive	[58]	N/A
232	Oc1ccccc1-c1[nH]c2[n+](ccnc2N)c1Nc1ccccc1	23_5	Inactive	[58]	N/A
233	OCCCn1c2c(nc1C[NH2+]CC)c1c(nc2)cccc1	6a_2011	Inactive	[56]	N/A
234	O(C)c1ccc(cc1)Cn1c2c(nc1-c1ccccc1)c1c(nc2)cccc1	5b_2011	Inactive	[56]	N/A

Table S2. List of 13 small molecular weight compounds selected for experimental validation.

#	Name	Compound ID	IUPAC name	Molecular weight (g/mol)
1	TAC1	ZINC2901940	(1-heptylbenzimidazol-3-ium-2-yl)amine	231
2	TAC2	ZINC95362363	5-ethyl-N4-[3-[(5-methyl-2-pyridyl)amino]propyl]pyrimidine-2,4-diamine	286
3	TAC3	ZINC04679422	1-butyl-5-(4-ethoxyphenyl)-imidazol-2-amine	259
4	TAC4	MolPort-019-730-277	N-(1,2,3,4-tetrahydroquinolin-2-ylidene)butan-1-amine	202
5	TAC5	MolPort-000-141-803	3-(benzyloxy)pyridin-2-amine	200
6	TAC6	MolPort-001-844-727	N2-benzyl-N4-butyl-6-methylpyrimidine-2,4-diamine	270
7	TAC7	MolPort-002-892-663	3-[(3-methylphenyl)methoxy]pyridin-2-amine	214
8	TAC8	MolPort-000-164-223	1-[2-(diethylamino)ethyl]-1H-1,3-benzodiazol-2-amine	232
9	TAC9	ZINC4070639	1-naphthaleneacetamide, N-(2-methyl-4-nitrophenyl)	320
10	TAC10	ZINC1388538	5,6-dimethyl-1-propyl-1H-1,3-benzimidazol-4-ylamine	203
11	TAC11	ZINC91914538	5-ethyl-N4-[3-(3-pyridyl)propyl]pyrimidine-2,4-diamine	257
12	TAC12	MolPort-001-886-853	12,14-dimethyl-17-thia-9,15-diazatetracyclo[8.7.0.0^{2},^{7}.0^{11},^{16}]heptadeca-1(10),2,4,6,8,11(16),12,14-octaen-8-amine	279
13	TAC13	MolPort-005-925-640	4-[2-(dimethylamino)ethyl]-5,6-dimethyl-2,4-diazatricyclo[7.5.0.0^{3},^{7}]tetradeca-1(9),2,5,7-tetraen-8-amine	300

Supplementary Methods

Chemical synthesis and characterization of TAC5 derivatives

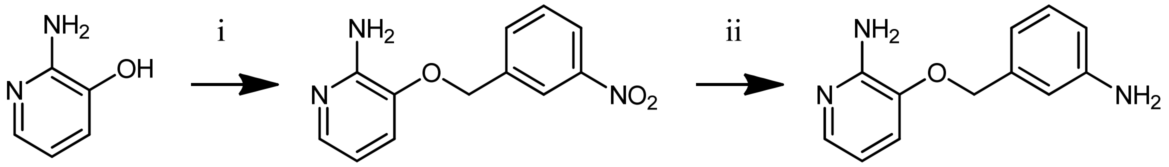

Synthesis of 3-((4-aminobenzyl)oxy)pyridin-2-amine (TAC5-a)

Reagents and conditions: (i) 3-nitrobenzylbromide, Cs_2CO_3, DMF, RT; (ii) $SnCl_2$, EtOH, reflux.

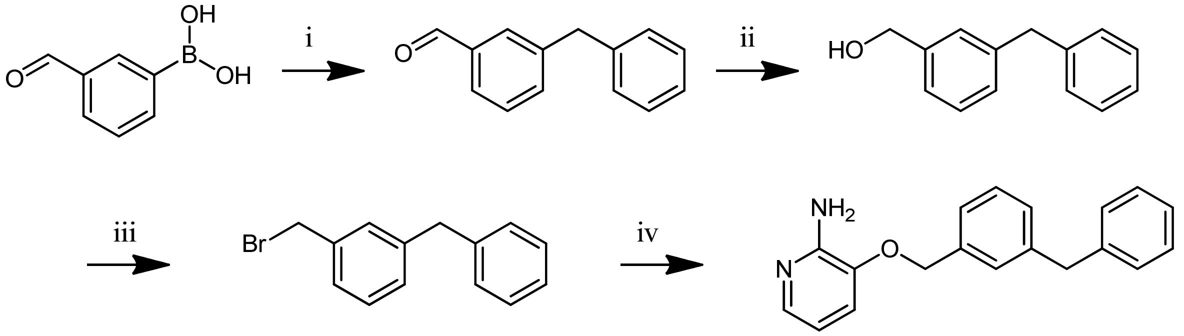

Synthesis of 3-((3-benzylbenzyl)oxy)pyridin-2-amine (TAC5-b)

Reagents and conditions: (i) benzylbromide, $Pd(PPh_3)_4$, Na_2CO_3, toluene, 80 °C; (ii) $NaBH_4$, Me OH, 0 °C; (iii) HBr, water:CH_2Cl_2 (v:v = 1:1), RT; (iv) 2-amino-3-hydroxylpyridine, Cs_2CO_3, DM F, RT.

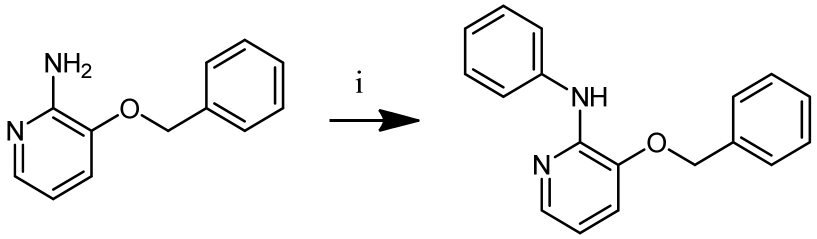

Synthesis 3-(benzyloxy)-N-phenylpyridin-2-amine (TAC5-c)

Reagents and conditions: (i) bromobenzene, CuI, DMEDA, K_2CO_3, 1,4-dioxane, 90 °C.

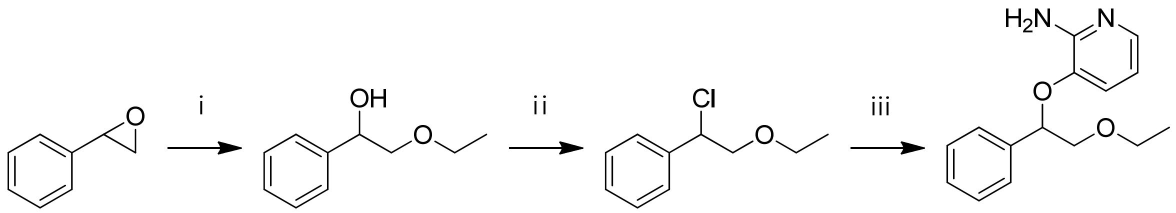

Synthesis of 3-(2-ethoxy-1-phenylethoxy)pyridine-2-amine (TAC5-d).

Reagent condition: (i) EtOH, NaOH, reflux; (ii) thionyl chloride, DCM, 0 °C to RT; (iii) Cs_2CO_3, 2-amino-3-hydroxylpyridine, DMF, RT.

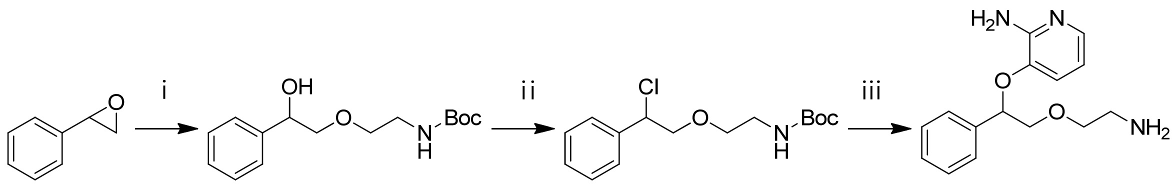

Synthesis of 3-(2-(2-aminoethoxy)-1-phenylethoxy)pyridin-2-amine (TAC5-e).

Reagents and conditions: (i) N-Boc-ethanolamine, NaH, THF, 60 °C; (ii) thionyl chloride, DCM, 0 °C; (iii) 1. Cs_2CO_3, 3-hydroxyl-2-aminopyridine, DMF, RT; 2. TFA, DCM (v:v = 1:2), RT, 4 h.

General chemistry methods

All reactions were carried out under an atmosphere of nitrogen or argon in air-dried glassware with magnetic stirring. Air- and/or moisture-sensitive liquids were transferred with a syringe. Organic solutions were concentrated by rotary evaporation at 25–60 °C at 15–30 torr. All solvents and common materials were purchased from suppliers and used without further purification. Column chromatography was carried out as “Flash Chromatography” using a Biotage MPLC machine. ^{1}H NMR and ^{13}C NMR data were recorded on a JEOL ECZ-600R Magnetic Resonance System (600 MHz) at Ajou University. Recorded chemical shifts were reported in parts per million (δ). Multiplicity was indicated as follows: s (singlet); d (doublet); t (triplet); q (quartet); m (multiplet); br (broad) etc. Coupling constants (J) were reported in Hz. Low-resolution mass spectrometry (LRMS) was obtained by an LC/MS system, Finnigan MSQplus Surveyer (Thermo Fisher Scientific, Inc, Waltham, MA, USA) or 6120 Quadrupole LC/MS (Agilent Technologies, Santa

Clara, CA, USA). All chemicals were purchased from Sigma-Aldrich, Co. (St. Louis, MO, USA), Tokyo Chemical Industry Co., Ltd (Tokyo, Japan) or ThermoFisher Scientific, Inc. and used without further purification unless otherwise specified. The reaction progress was monitored using thin-layer chromatography (TLC) (silica gel 60, F254 0.25 mm), and components were visualized by observation under UV light (254 and 365 nm) or by treating the TLC plates with phosphomolybdic acid (PMA), $KMnO_4$, or ninhydrin followed by heating.

Synthesis

Synthesis of 3-((3-nitrobenzyl)oxy)pyridin-2-amine (TAC5-a intermediate)

3-Nitrobenzyl bromide (4.0 g, 18.52 mmol) in DMF (20 ml) was added to a solution of 2-amino-3-hydroxyl pyridine (2.04 g, 18.52 mmol) and Cs_2CO_3 (6.03 g, 18.52 mmol) in DMF (80 ml), stirred in an argon atmosphere and the resulting reaction mixture was stirred at room temperature for 3 h. After reaction completion, the reaction mixture was added to a saturated $NaHCO_3$ (aq.) solution and extracted with CH_2Cl_2 three times. The combined organic layer was dried with Na_2SO_4 (s) and concentrated *in vacuo*. The crude product was purified with silica-gel column chromatography to yield a product (2.20 g, yield = 48.4%) as a yellow solid. ^{1}H NMR (600 MHz, $CDCl_3$) δ 8.29 (s, 1H), 8.21 (dd, J = 8.1 Hz, 1H), 7.76 (d, J = 7.7 Hz, 1H), 7.70 (d, J = 4.8 Hz, 1H), 7.59 (t, J = 7.9 Hz, 1H), 6.95 (dd, J = 7.8 Hz, 1H), 6.60 (dd, J = 7.8 Hz, 1H), 5.12 (s, 2H), 4.75 (bs, 2H); ^{13}C NMR (150 MHz, $CDCl_3$) δ 150.2, 148.5, 141.0, 139.9, 138.5, 133.4, 129.9, 123.4, 122.4, 117.0, 113.7, 69.0.

Synthesis of 3-((3-aminobenzyl)oxy)pyridin-2-amine (TAC5-a)

A mixture of Tin (II) chloride (7.732 g), 1 N HCl (aq, 0.2 ml), distilled water (40 ml), and 3-((3-nitrobenzyl)oxy)pyridin-2-amine (2.0 g) was refluxed for 3 h. After reaction completion, the reaction mixture was cooled to room temperature. The resulting precipitated solid was collected by filtration, dissolved with saturated $NaHCO_3$ (aq.) solution and extracted with EA) three times. The combined organic layer was dried with Na_2SO_4 (s) and concentrated *in vacuo* to yield a product (945 mg, yield = 53.9%) as a white solid without further purification. ^{1}H NMR (600 MHz, $CDCl_3$) δ 7.67 (d, J = 4.2 Hz , 1H), 7.17 (t, J = 7.8 Hz, 2H), 6.94 (d, J = 8.4 Hz, 1H), 6.73(s, 1H), 6.66(dd, J = 8.1 Hz, 1H), 6.58(dd, J = 7.8Hz, 1H), 4.98(s, 2H), 4.70(bs, 2H), 3.72(bs, 2H); ^{13}C NMR (150 MHz, $CDCl_3$) δ 150.3, 146.8, 141.6, 139.1, 137.6, 129.7, 117.6, 116.9, 115.0, 113.9, 113.8, 70.3.. LRMS (ESI) m/z calculated for $C_{12}H_{14}N_3O$ $[M+H]^+$: 216.11; found: 216.00.

Synthesis of 3-benzylbenzaldehyde (TAC5-b intermediate 1)

3-Formyl phenylboronic acid (450 mg, 3.0 mmol) and tetrakis(triphenylphosphine)palladium(0) (139 mg, 0.12 mmol) were added and purged with Ar. Toluene (15 ml), benzyl bromide, and 2 N Na_2CO_3 (3 ml) were added and the resulting reaction mixture was stirred at 80 °C for 4 h. After reaction completion, the reaction mixture was diluted with $NaHCO_3$ and extracted with CH_2Cl_2 three times. The combined organic layer was concentrated *in vacuo*. The crude product was purified by silica-gel column chromatography (EA:Hex = Hex 100%-EA 15%) to yield a product (503 mg, yield = 85.3%) as a clear oil. ^{1}H NMR (600 MHz, $CDCl_3$) δ 9.86 (s, 1H), 7.63 (m, 2H), 7.23 (m, 7H), 3.95 (s, 2H); ^{13}C NMR (150 MHz, $CDCl_3$) δ 191.9, 141.9, 139.8, 136.4, 134.7, 129.5, 128.8, 128.6, 128.4, 127.4, 126.1, 41.2.

Synthesis of (3-Benzylphenyl)methanol (TAC5-b intermediate 2)

$NaBH_4$ (75 mg, 2 mmol) was added dropwise to a solution of 3-benzylbenzaldehyde (196 mg, 1.0 mmol) in methanol (10 ml) that was stirred at 0 °C. The resulting reaction mixture was allowed to stir at 0 °C for 1 h. After reaction completion, the reaction mixture was washed with distilled water and extracted with CH_2Cl_2 three times. The combined organic layer was concentrated *in vacuo*. The crude product was purified with silica-gel column chromatography (EA:Hex = Hex 100%-EA 50%) to yield a product (203 mg, quantitative yield) as a clear oil. ^{1}H NMR (600 MHz, $CDCl_3$) δ 7.13 (m, 9H), 4.41 (s, 2H), 3.89 (s, 2H), 2.89 (s, 1H); ^{13}C NMR (150 MHz, $CDCl_3$) δ 141.2, 140.9, 140.9, 128.7, 128.4, 128.3, 127.9, 127.3, 125.9, 124.6, 64.7, 41.7.

Synthesis of 1-benzyl-3-(bromomethyl)benzene (TAC5-b intermediate)

(3-Benzylphenyl)methanol (198 mg, 1.0 mmol) was diluted with a 1:1 (v/v) mixture of DCM and HBr mixture (5 ml), and the resulting reaction mixture was stirred at room temperature for 36 h. After reaction completion, the reaction mixture was diluted with $NaHCO_3$ and extracted with CH_2Cl_2 three times. The combined organic layer was concentrated *in vacuo*. The crude product was purified using silica-gel column chromatography (EA:Hex = Hex 100%-EA 10%) to yield a product (185 mg, yield = 70.8%) as a clear oil. ^{1}H NMR (600 MHz, $CDCl_3$) δ 7.17 (m, 9H), 4.36 (s, 2H), 3.92 (s, 2H); ^{13}C NMR (150 MHz, $CDCl_3$) δ 141.6, 140.5, 137.8, 129.4, 129.0, 128.9, 128.8, 128.5, 126.8, 126.1, 41.6, 33.5.

Synthesis of 3-((3-benzylbenzyl)oxy)pyridin-2-amine (TAC5-b)

1-Benzyl-3-(bromomethyl)benzene (178 mg, 0.68 mmol) in DMF (3.8 ml) was added to a solution of 2-amino-3-hydroxy pyridine (75 mg, 0.68 mmol) and Cs_2CO_3 (222 mg, 0.68 mmol) in DMF (3 ml). The resulting reaction mixture was stirred at room temperature for 2 h. After reaction completion, the reaction mixture was diluted with $NaHCO_3$ and extracted with CH_2Cl_2 three times. The combined organic layer was concentrated *in vacuo*. The crude product was purified using silica-gel column chromatography (EA:Hex = Hex 100%-EA 100%) to yield a product (110 mg, yield = 55.9%) as a yellow solid. ^{1}H NMR (600 MHz, $CDCl_3$) δ 7.65 (d, J = 6.0 Hz, 1H), 7.21 (s, 9H), 6.88 (d, J = 6.6 Hz, 1H), 6.53 (dd, J = 7.8 Hz, 1H), 4.94 (s, 2H), 4.79 (bs, 2H), 3.96 (s, 2H); ^{13}C NMR (150 MHz, $CDCl_3$) δ 150.3, 141.5, 141.3, 140.6, 138.9, 136.5, 128.8, 128.7, 128.6, 128.4, 128.0, 126.1, 125.2, 116.7, 113.3, 70.0, 41.6; LRMS (ESI) m/z calculated for $C_{19}H_{19}N_2O$ $[M+H]^+$: 291.14; found: 291.20.

Synthesis of 3-(benzyloxy)-N-phenylpyridin-2-amine (TAC5-c)

A solution of bromobenzene (786 μl, 7.5 mmol) was added slowly to a solution of 2-amino-3-benzyloxypyridine (1.0 g, 5.0 mmol), copper (I) iodide (0.48 g, 2.5 mmol), potassium carbonate (1.38 g, 10.0 mmol), and *N,N'*-dimethylethylenediamine (270 μl, 2.5 mmol) in 1,4-dioxane (25 ml),. The resulting reaction mixture was stirred at 90 °C for 17 h. After reaction completion, the reaction mixture was diluted with saturated $NaHCO_3$ (aq.) and extracted with CH_2Cl_2 three times. The combined organic layer was concentrated *in vacuo*. The crude product was purified with silica-gel column chromatography (EA:Hex = Hex 100%-EA 20%) to yield a product (0.11 g, yield = 15.8% (isolation yield)) as a white solid. ^{1}H NMR (600 MHz, $CDCl_3$) δ 7.86 (dd, J = 4.8 Hz , 1H), 7.70 (d, J = 7.8 Hz, 2H), 7.46-7.39 (m, 5H), 7.23 (t, J = 7.8 Hz, 2H), 7.07 (bs, 1H), 7.04 (dd, J = 7.8 Hz, 1H), 6.69 (dd, J = 7.4 Hz, 1H), 5.14 (s, 2H); ^{13}C NMR (150 MHz, $CDCl_3$) δ 147.0, 141.7, 141.0, 138.9, 136.4, 129.2, 129.1, 128.7, 128.0, 121.9, 119.2, 116.6, 114.4, 70.7. LRMS (ESI) m/z calculated for $C_{18}H_{17}N_2O$ $[M+H]^+$: 277.13; found: 277.15.

Synthesis of 2-ethoxy-1-phenylethanol. (TAC5-d intermediate 1)

To a solution of NaOH (440 mg, 11 mmol) in EtOH (50 ml), stirred under Ar atmosphere, wad added styrene oxide (1.13 ml, 10 mmol) and resulting reaction mixture was refluxed for 3 h. Reaction mixture was cooled down to RT, diluted with sat. $NaHCO_3$ (aq.) and extracted with DCM for three times. Combined DCM layer was dried over Na_2SO_4 (s) and concentrated *in vacuo*. Flash silica gel column chromatography afforded the desired product (800 mg, 48.1 %) as a clear oil. 1H

NMR (600 MHz, $CDCl_3$) δ 7.36 (m, 5H), 4.88 (dd, J = 9.6 Hz, 1H), 3.57 (m, 3H), 3.43 (t, J = 9.0 Hz, 1H), 3.14 (s, 1H), 1.23 (t, J = 6.6 Hz, 3H); 13C NMR (150 MHz, $CDCl_3$) δ 140.3, 128.2, 127.7, 126.1, 76.1, 72.7, 66.6, 15.0.

Synthesis of (1-chloro-2-ethoxyethyl)benzene. (TAC5-d intermediate 2)

To a solution of compound 4-a (1.0 g, 6.02 mmol) in DCM (15 ml), stirred at 0 °C, added a solution of thionyl chloride (524 µl, 7.22 mmol) in DCM (10 ml) and the resulting reaction mixture was stirred at 0 °C. After stirring at 0 °C for an additional 1 h, the reaction mixture was gradually warmed up to RT. After reaction completion, the reaction mixture was diluted with $NaHCO_3$ (aq.) and extracted with DCM three times. The combined DCM layer was dried over Na_2SO_4 (s) and concentrated *in vacuo*. Flash silica gel column chromatography afforded the desired product (452 mg, 40.7 %) as a clear oil. ^1H NMR (600 MHz, $CDCl_3$) δ 7.36 (m, 5H), 4.99 (dd, J = 7.2 Hz, 1H), 3.81 (m, 2H), 3.55 (m, 2H), 1.97 (t, J = 7.2 Hz, 3H); ^{13}C NMR (150 MHz, $CDCl_3$) δ 138.7, 128.4, 128.3, 127.2, 75.1, 66.5, 61.0, 14.8.

Synthesis of 3-(2-ethoxy-1-phenylethoxy)pyridine-2-amine (TAC5-d)

To a solution of 2-amino-3-hydroxypyridine (120 mg, 1.08 mmol) and KOH (68 mg, 1.08 mmol) in DMF (5 ml) was added compound 4-b (200 mg, 1.08 mmol) and resulting reaction mixture was stirred at 60 °C for 4 h. After reaction completion, reaction mixture was diluted with $NaHCO_3$ (aq.) and extracted with DCM for three times. Combined DCM layer was dried over Na_2SO_4 (s) and concentrated *in vacuo*. Flash silica gel column chromatography afforded the desired product (137 mg, 49.1 %) as a white solid. ^1H NMR (600 MHz, $CDCl_3$) δ 7.65 (dd, J = 6.0 Hz, 1H), 7.36 (m, 5H), 6.71 (d, J = 8.4 Hz, 1H), 6.39 (dd, J = 7.8 Hz, 1H), 5.12 (dd, J = 9.0 Hz, 1H), 5.02 (bs, 2H), 3.84 (m, 1H), 3.61 (m, 3H), 1.24 (t, J = 7.2 Hz, 3H) ; ^{13}C NMR (150 MHz, $CDCl_3$) δ 151.7, 140.4, 140.3, 137.9, 128.5, 128.1, 126.3, 121.7, 112.9, 81.5, 74.7, 66.7, 14 .9; LRMS (ESI) m/z calcd. for $C_{15}H_{19}N_2O_2$ [M+H]+ 258.14; found : 259.05.

Synthesis of tert-butyl (2-(2-hydroxy-2-phenylethoxy)ethyl)carbamate (TAC5-e intermediate 1)

To a solution of NaH (60 % dispersion in mineral oil, 40 mg, 1.0 mmol) in *N*-Boc-ethanolamine : THF (1.0 ml, v:v = 1:1) was stirred at 60 °C, added styrene oxide (114 µl, 1.0 mmol) and resulting reaction mixture was stirred at 60 °C for overnight. After reaction completion, reaction mixture

was diluted with $NaHCO_3$ (aq.) and extracted with DCM for three times. Combined DCM layer was dried over Na_2SO_4 (s) and concentrated *in vacuo*. Flash silica gel column chromatography afforded the desired product (70 mg, 24.9 %) as a clear oil.; ^1H NMR (600 MHz, $CDCl_3$) δ 7.36 (m, 5H), 5.04 (bs, 1H), 4.87 (dd, J = 8.4 Hz, 1H), 3.58 (m, 4H), 3.32 (bs, 2H), 3.22 (s, 1H), 1.43 (s, 9H); ^{13}C NMR (150 MHz, $CDCl_3$) δ 140.3, 128.3, 127.8, 126.1, 76.5, 72.7, 70.4, 40.3, 28.4.

Synthesis of tert-butyl (2-(2-chloro-2-phenylethoxy)ethyl)carbamate (TAC5-e intermediate 2)

To a solution of compound 5-a (128 mg, 0.46 mmol) in DCM (4.5 ml), stirred at 0 °C, added thionyl chloride (40 µl, 0.55 mmol) and resulting reaction mixture was stirred at 0 °C for 1.5 h. After reaction completion, reaction mixture was diluted with $NaHCO_3$ (aq.) and extracted with DCM for three times. Combined DCM layer was dried over Na_2SO_4 (s) and concentrated *in vacuo*. Flash silica gel column chromatography afforded the desired product (69 mg, 50.6 %) as a clear oil.; ^1H NMR (600 MHz, $CDCl_3$) δ 7.36 (m, 5H), 4.97 (t, J = 6.0 Hz, 1H), 4.79 (s, 1H), 3.86 (m, 2H), 3.55 (m, 2H), 3.27 (s, 2H), 1.44 (s, 9H); ^{13}C NMR (150 MHz, $CDCl_3$) δ 138.5, 128.7, 128.6, 127.4, 75.5, 70.3, 60.8, 40.2, 28.4.

Synthesis of 3-(2-(2-aminoethoxy)-1-phenylethoxy)pyridin-2-amine (TAC5-e)

A solution of compound 5-b (69 mg, 0.24 mmol), Cs_2CO_3 (79 mg, 0.24 mmol), and 2-amino-3-hydroxyl pyridine (27 mg, 0.24 mmol) in DMF (3 ml) was stirred at RT to 40 °C for overnight. After reaction completion, reaction mixture was diluted with $NaHCO_3$ (aq.) and extracted with DCM for three times. Combined DCM layer was dried over Na_2SO_4 (s) and concentrated *in vacuo*. Flash silica gel column chromatography afforded the desired product. Purified product was used for next reaction without further characterization. Purified compound was stirred in DCM:TFA (v:v = 2:1) at RT for 4 h. After reaction completion, reaction mixture was diluted with $NaHCO_3$ (aq.) and extracted with DCM for three times. Combined DCM layer was dried over Na_2SO_4 (s). Concentration of resulting filtrate afforded the desired product (10 mg, 15.2 %) as a yellow solid.; ^1H NMR (600 MHz, CD_3OD) δ 7.43 (m, 6H), 8.86 (d, J = 7.8 Hz, 1H), 6.42 (dd, J = 7.8 Hz, 1H), 5.39 (dd, J = 7.2 Hz, 1H), 3.93 (dd, J = 10.8 Hz, 1H), 3.75 (dd, J = 6.6 Hz, 1H), 3.61 (t, J = 5.4 Hz, 2H), 3.35 (s, 1H), 2.81 (bs, 2H); ^{13}C NMR (150 MHz, CD_3OD) δ 152.9, 142.1, 139.4, 139.2, 129.7, 129.4, 127.7, 121.3, 113.9, 81.5, 76.2, 73,4, 41.9.; LRMS (ESI) m/z calcd. for $C_{15}H_{20}N_3O_2$ $[M+H]^+$ 274.15; found : 274.10.
